# Defining variation in pre-human ecosystems can guide conservation: An example from a Caribbean coral reef

**DOI:** 10.1038/s41598-020-59436-y

**Published:** 2020-02-19

**Authors:** Aaron O’Dea, Mauro Lepore, Andrew H. Altieri, Melisa Chan, Jorge Manuel Morales-Saldaña, Nicte-Ha Muñoz, John M. Pandolfi, Marguerite A. Toscano, Jian-xin Zhao, Erin M. Dillon

**Affiliations:** 1 Smithsonian Tropical Research Institute, Box 0843-03092, Balboa, Republic of Panama; 2 Department of Biological, Geological and Environmental Sciences, University of Bologna, Piazza Porta San Donato 1, 40126 Bologna, Italy; 3 Department of Environmental Engineering Sciences, University of Florida, Gainesville, FL 32611 USA; 4 Australian Research Council Centre of Excellence for Coral Reef Studies, School of Biological Sciences, University of Queensland, St. Lucia, Queensland 4072 Australia; 5 Smithsonian Environmental Research Center, 647 Contees Wharf Road, Edgewater, Maryland 21037 USA; 6 School of Earth & Environmental Sciences, University of Queensland, St. Lucia, Queensland 4072 Australia; 7 Department of Ecology, Evolution and Marine Biology and the Marine Science Institute, University of California, Santa Barbara, CA 93106 USA

**Keywords:** Conservation biology, Palaeoecology, Marine biology, Community ecology, Conservation biology, Ecosystem ecology, Tropical ecology

## Abstract

Many Caribbean coral reefs are heavily degraded, yet their pre-human, natural states are often assumed or estimated using space-for-time substitution approaches. Here we use an 11-hectare suite of fossilised mid-Holocene (7.2–5.6 ka) fringing reefs in Caribbean Panama to define natural variation in hard coral community structure before human-impact to provide context to the states of the same reefs today. We collected bulk samples from four trenches dug into the mid-Holocene fossil reef and surficial bulk samples from 2–10 m depths on five adjacent modern reefs extending over 5 km. Analysis of the abundances of coral taxa in fossil bulk samples define the Historical Range of Variation (HRV) in community structure of the reefs. When compared to the community structure of adjacent modern reefs, we find that most coral communities today fall outside the HRV, identifying them as novel ecosystems and corroborating the well-documented transition from acroporid-dominated Caribbean reefs to reefs dominated by stress-tolerant taxa (*Porites* and *Agaricia*). We find one modern reef, however, whose community composition remains within the HRV showing that it has not transitioned to a novel state. Reef-matrix cores extracted from this reef reveal that the coral community has remained in this state for over 800 years, suggesting long-term stability and resistance to the region-wide shift to novel states. Without these data to provide historical context, this potentially robust and stable reef would be overlooked since it does not fulfil expectations of what a Caribbean coral reef should look like in the absence of humans. This example illustrates how defining past variation using the fossil record can improve our understanding of modern degradation and guide conservation.

## Introduction

Caribbean coral reefs began to deteriorate long before they were first surveyed^1–7^. Consequently, modern benchmarks used to inform and evaluate the success of conservation actions rely on space-for-time substitution—an approach that uses spatial patterns across modern ecosystems as a proxy for temporal changes within ecosystems. The space-for-time substitution approach is a mainstay across conservation ecology, and has previously proved effective in coral reef ecology^8^, but the assumption that spatial and temporal variation are equivalent may not necessarily be true^9^ especially in habitats that are naturally highly variable on small spatial and temporal scales, like coral reefs^10^. Conditions from one reef therefore cannot necessarily be used to define another’s baseline. Additionally, with so few reconstructions of the state of reefs before human impact, baselines rely on limited definitions of reef states that likely fail to capture the high spatial and temporal complexity of coral reefs^3,11^. Accordingly, to prioritize conservation activities, contextualise restoration goals, and reveal the drivers of ecosystem change, it is important to better quantify spatial and temporal variation on Caribbean reefs, both past and present.

**Figure 1:**
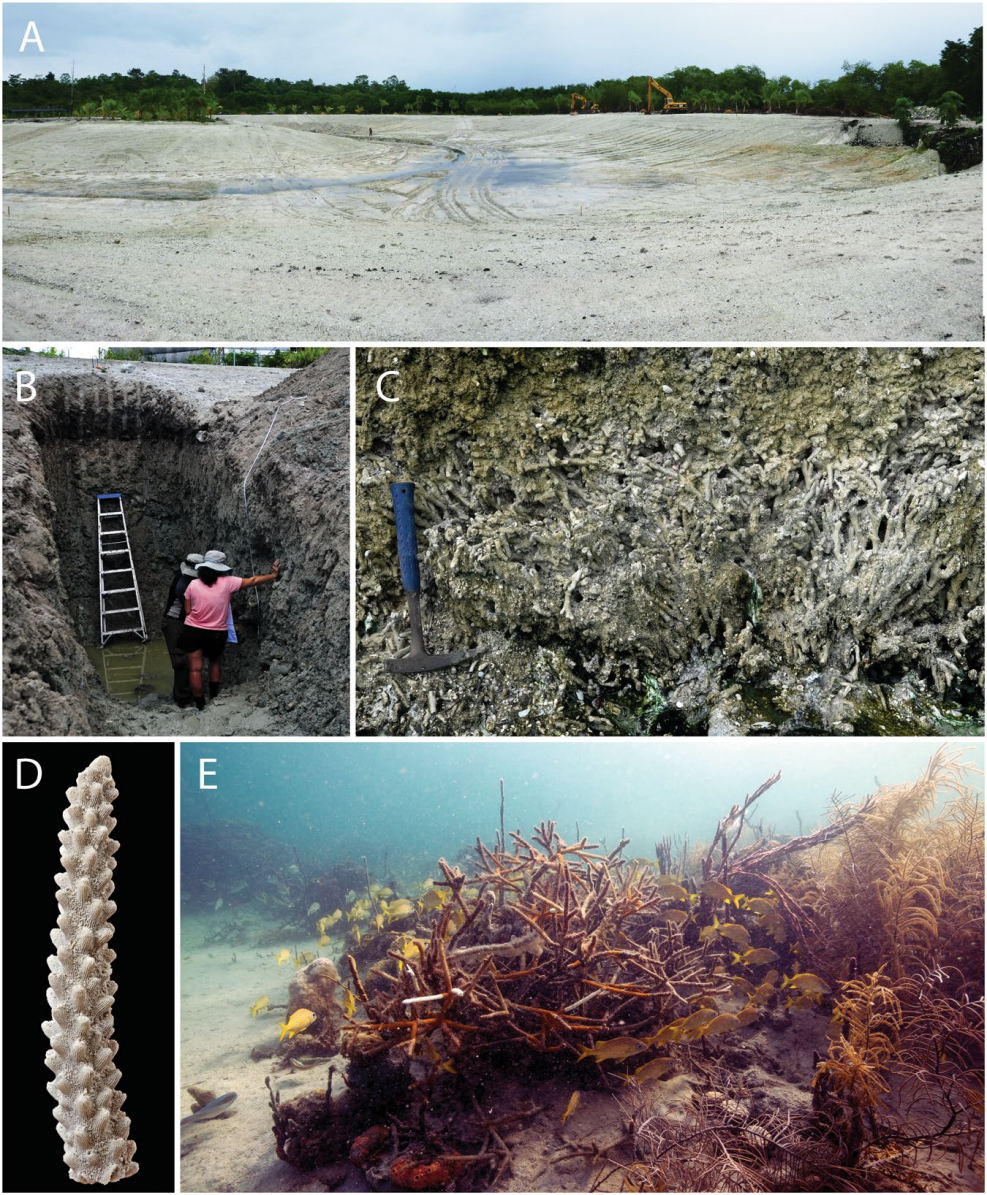
Fossil and modern coral reefs of eastern Almirante Bay, Caribbean Panama. (**A**) Large-scale excavation revealed the mid-Holocene fossil coral reef in Bocas del Toro, where we dug four trenches (**B**) to expose the autochthonous and in life position (**C**) fossil reef for bulk sampling. Preservation of corals, like *A. cervicornis* was excellent (**D**). The coral community composition at the modern reef at Punta Caracol (**E**) was found to be encapsulated by the variation described in the mid-Holocene reefs. Images courtesy of Harry Taylor (**D**) and David Kline (**E**).

**Figure 2:**
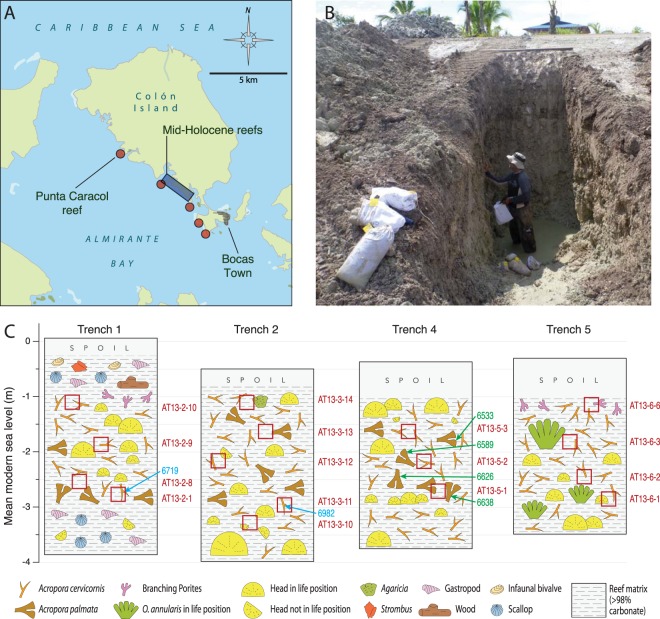
(**A**) Map of study area showing the location of modern reefs sampled (red circles) and known extent of mid-Holocene reef (blue rectangle). Map is centered on 9.359395 N, 82.319180 W. (**B**) Example of the Trenches (#4) in the mid-Holocene reef made to permit bulk sampling of *in situ* reef framework. (**C**) Stratigraphic sections of the trenches showing dominant taxa and positions of bulk samples (red squares), U-Th dates (blue) and radiocarbon dates (green), both years BP.

The Historical Range of Variation (HRV) is an approach developed in the field of terrestrial ecology^12,13^ that uses paleontological and historical records to define past spatial and temporal variation in ecosystem states with the aim of helping to determine if current states and rates of change fall within or outside the natural range of variation^14,15^. Coral reefs are potentially well-suited to the application of this approach given their high community complexity, and their propensity to preserve a wide array of reef community members^16–24^ and past environmental signals^25^ as they accrete. In this study, we apply the HRV approach to quantify variation in pre-human coral communities in a suite of lagoonal fringing coral reefs in western Caribbean Panama (Figs. 1 and 2) using ~7000-year-old (mid-Holocene) fossil assemblages. We then compare the HRV with the variation that exists on adjacent modern reefs today to quantify modern day ecosystem states within their historical context. Our findings provide a case study with which to discuss what should be considered natural and novel in ecosystems such as Caribbean coral reefs that are experiencing rapid changes.

## Materials and Methods

### General setting

The fringing coral reefs, seagrass and mangrove systems of Almirante Bay (Fig. 2A) became established shortly after the bay flooded around 8000 years ago when sea levels rose^18^ and have persisted since^1,26,27^. Today, the reefs fringing the bay are primarily composed of stress-tolerant and “weedy” corals like branching *Porites* and *Agaricia*^28,29^, but it hasn’t been demonstrated if this represents the natural state of reefs in the bay. Coral cover is generally moderate to low and fish communities are depauperate of adults^30^. Almirante Bay is well protected from wave action and currents, and sits outside the hurricane belt. High rainfall, deforestation and changing land use, including a ~140-year banana industry^31^, cause relatively higher suspended particles and dissolved nutrients conditions compared to other Caribbean reef systems^32,33^. The bay today experiences periodic hypoxia that sometimes causes widespread death of corals and other aerobic sessile organisms^34,35^.

### The fossil reef

Two large-scale construction projects led to the excavation of ~50 hectares of a fossil fringing coral reef in the eastern portion of Almirante Bay (Figs. 1 and 2). Previous Uranium-Thorium (U-Th) dates from coral pieces placed the fossil reefs in the mid-Holocene, ~7 ka^18^, a date which we confirm in this study (see later).

The mid-Holocene was a time of environmental change both globally and in the Tropical Western Atlantic region, associated with warming, ENSO variability and shifts in the location of the intertropical convergence zone^36^. Paleotemperature reconstructions of the Caribbean at this time are equivocal, suggesting either mid-Holocene warming^37^ or cooling^38^ with greater seasonality^39^ relative to today. Most of these reconstructions relate to regions to the north and eastern Caribbean region. Estimates from the southwestern Caribbean of Panama where our study is based are limited, but available models indicate similar climatic conditions to today^39^. Other available evidence suggests the fossil reef formed in a setting similar to the reefs of today. Post-glacial sea level rise had stabilised close to modern day levels as the fossil reef accreted^40,41^, a result corroborated by our elevation models, similarities between coral (see later) and the structure of fish communities in the reefs as observed through fossil otolith assemblages^21^. Reefs in the region today sit to the south of the hurricane belt and this appears to have also been the case for at least the last 7 ka^42^. The aspect and exposure of the fossil reef was the same as it is today given that the Island of Colón, which today forms the Almirante bay (Fig. 2), is composed of Plio-Pleistocene sediments and would therefore have formed a similar protective barrier and lagoonal setting as it does today. The presence of thick lagoonal muds and mangrove peats in the fossil reef that accumulate only in such protected settings^18^ corroborates this conclusion.

Humans were on the Isthmus of Panama before 14 ka^43,44^ but remained concentrated on the Pacific coast until major settlements of people exploiting Caribbean marine resources appeared after 4 ka^45–47^. As such, this large fossil coral reef offers an opportunity to quantitatively reconstruct the spatial and temporal variation in community structure in a fringing reef system from a time before major human impact when environmental conditions and settings were comparable to today.

### Quantifying the structure of mid-Holocene and modern coral communities

We mapped ~11 hectares of the known 50 hectare extent of the mid-Holocene reef site using high-resolution Trimble R8 Base and Rover GNSS system to build a 3D model of the site (called Sweet Bocas) and locate major fossil habitats (see Fig. S1, for details). Using a large excavator, we dug four 4–5 m-deep and ~1.5 m-wide trenches into the reef to expose fresh *in situ* reef framework (Figs. 1 and 2; Fig. S2). Sections were measured and mapped and the vertical positions of both bulk samples and coral samples for dating were measured using the Trimble R8 system (Fig. S1). All elevations were adjusted to mean sea level (MSL) by measuring sea level directly adjacent to the fossil site and relating that to data from the tide gauge at the Smithsonian Tropical Research Institute’s Bocas del Toro Research Station.

To quantify the HRV in coral communities in the mid-Holocene fossil reefs, we extracted 16 bulk samples (~10 kg each) of reef framework covering a stratigraphic depth of ~15 cm from each of the four trenches we excavated (Figs. 1B and 2B). To compare the resulting HRV with modern reef communities, we collected 22 bulk samples (~10 kg each) of reef framework from the top <10 cm of the death assemblage from five modern fringing reefs adjacent to the fossil site (Fig. 1E). These modern death assemblage samples were collected at water depths between 2 and 10 m to ensure we covered the full range of depths sampled in the mid-Holocene. We are confident the depth of reef formation in the mid-Holocene was within this range because of the known depth range of *Acropora palmata* present in most sections (Fig. S3; The subjects in this figure both provided informed consent to publish in an open-access, online publication), the current elevation of the fossil reefs relative to sea level (Fig. 2), the distribution of fossil mangrove, seagrass and reef crest habitats as reported in Fredston-Hermann *et al*.^18^, Lin *et al*.^21^, and observed in this study, and finally results from modern and fossil foraminifera assemblages showing the modern and fossil reefs are comparable in environmental setting^48,49^.

In both mid-Holocene and modern bulk samples, all coral skeletal remains in the >2 mm fraction were identified to species or genus using the Caribbean Coral Skeleton Identification Guide^50^ – a purpose-built reference collection – and other guides^51^. The relative mass of each coral taxon was square-root transformed and used to compare the assemblage structure of each sample using Bray-Curtis dissimilarities, which were ordinated using non-metric multidimensional scaling (NMDS) to place samples in multivariate ecological space based on the relative abundance of coral taxa skeletal weights^50^. *PERMANOVA* was used to test for significant differences in dissimilarities among groups. Corals of unknown taxa were removed from all analyses. These corals are listed as “unknown” in Table S2, and were generally fragments identified as coral but too eroded or fragmented to be able to be confidently placed in a taxon^50^. “Unknown” corals totalled 8.26% of all coral weights in mid-Holocene samples and 10.02% in modern samples (Table S2), suggesting no substantial difference in preservation between the two ages. Relative weights of coral taxa in each sample were square-root transformed to reduce the influence of dominant taxa prior to analysis. To calculate Bray-Curtis dissimilarities of samples we used the function *vegdist* in the software package *vegan*^52^ in *R*. The NMDS (Kruskal’s Non-metric Multidimensional Scaling) was computed using the *isoMDS* function in the software package *MASS*^53^ in *R* which was then plotted using *ggplot*^54^ in *R*^55^. The function *adonis* (Permutational Multivariate Analysis of Variance Using Distance Matrices) in the software package *vegan*^52^ was used to test for significant differences in dissimilarities among groups. To confirm that differences between communities were valid, and not an artefact of differences between groups in how much each community deviates from the centroid of the group we used beta-dispersion with the *betadisper* function [Multivariate homogeneity of groups dispersions (variances)] in the software package *vegan*^52^. All analyses were performed in *R* (R core team).

### Chronological framework

We used both U-Th and radiocarbon radiometric dating to provide temporal context to the community composition data in both the fossil and modern reefs. To determine how old and for how long the mid-Holocene reef grew, we used calibrated radiocarbon dating on eight pieces of *Acropora palmata* and U-Th on two branches of *A. cervicornis* (Table S1) and combined these results with nine U-Th dates previously conducted on large coral heads from the same site^18^, resulting in a total of 19 dates. To estimate how much time is encapsulated in a modern bulk sample we applied U-Th dating on six pieces of *A. cervicornis* randomly selected from one of the bulk samples from the modern reef (i.e., a death-assemblage) at Punta Caracol reef. See supplementary materials for further information on radiometric dating methods.

## Results and Discussion

### Preservation and environmental setting

Corals from the mid-Holocene fringing reefs were found to be, on the whole, very well-preserved with chemically-pristine skeletal material^18^ (Fig. 1D). The proportion of colonies eroded to the point that they were unidentifiable, a proxy for taphonomic preservation, was similar in the modern (10.02%) and fossil reefs (8.26%), suggesting that selective dissolution of corals did not affect observed differences in community composition, or that the fossil assemblages had experienced greater taphonomic processes than the modern reefs. Indeed, many fossil coral colonies, even delicately branching forms, were found exquisitely preserved in life-position in unsorted fine carbonate muds and silts (Figs. 1 and 2; Fig. S2) demonstrating exceptional preservation with little physical disturbance. Across the 50 hectare fossil reef, distinct pockets of mangrove peats and seagrass muds were preserved in positions that would be expected given the local topography, and there were no sedimentary structures or evidence that would suggest any of the sediments had been storm-transported (Fig. S2)^18^ showing that these fossil reefs, and their associated coral, mangrove and seagrass communities are autochthonous assemblages that accumulated without major disturbance and no post-mortem transport.

### Age, accretion rates and time-averaging

U-Th and radiocarbon dating show that the trenches we excavated into the fossil reef accreted, without apparent interruption, over a period of at least 1,600 years (7.2-5.6 ka) (Table S1), a time when sea-level had reached similar levels to today. Based on the elevation and radiocarbon dates of *Acropora palmata* colonies in Trench 4 (Fig. 2), estimated accretion rates of the fossil reef were on the order of around 10 mm year^−1^. This rate is almost identical to estimates from other prehistorical Caribbean reefs^56^ as well as *Acropora*-dominated reefs in the west Pacific^57^, but around four-times faster than estimates of accretion from post-european and post industrial aged reefs in the Almirante Bay that are now accreting at around 2.5 mm year^−1^ ^20^.

To estimate the amount of time encapsulated in a bulk sample, U-Th dates from a bulk sample taken from one of the modern reefs (Punta Caracol) ranged in age from 1926 to 2012 AD (mean = 1991 AD, SD = 30.4 years, n = 6; Table S1), suggesting that when they are actively accreting, coral assemblages in reefs with branching framework are predominantly formed by modern corals (i.e., post 1950). This finding supports previous work showing little vertical mixing in reef matrices that are composed of branching corals that accumulate in protected areas^20,21,58^, presumably because the branching coral acts as a rigid framework that limits infaunal and epifaunal bioturbation which can otherwise cause considerable vertical mixing in other settings^59,60^. These findings, along with the high frequency of fragile branching corals found in life position in the fossil reef (Figs. 1C and 2C, Fig. S2), reinforces the conclusion that the Almirante Bay has remained protected from major physical disturbance over the last 7000 years, and that the coral assemblages are autochthonous.

Death assemblages sampled through bulk-sampling are not directly comparable to traditional reef survey methods such as live coral cover. Variable rates of carbonate production and potential ephemeral growth patterns of different coral taxa will offset the relative abundances between traditional survey methods and abundances in skeletal records. In some cases, however, the contribution to skeletal framework in death assemblages can be remarkably constant across different taxa and different growth modes, and accretion rates are instead controlled more by local environmental and biotic conditions on the reef^19,20,57,61–63^. What is important, however, is that our samples across time were made as ecologically-comparable as possible by bulk-sampling death assemblages on the living reefs in the same way the fossil reefs were sampled. Based on estimated accretion rates of ~10 mm year^-1^, the fossil bulk samples in our study will incorporate ~150 years of time and based on U-Th analyses our modern bulk samples incorporate at least 90 years of time suggesting they are roughly comparable in the amount of time they incorporate. Assuming, therefore, that taphonomic processes are affecting the fossil and modern samples equally (see previously), the abundances of coral taxa in modern and fossil bulk samples yield meaningful and comparable ecological signals that relate to the relative contribution of different taxa in a time-averaged living community.

### Coral community composition in the mid-Holocene and today

**Figure 3:**
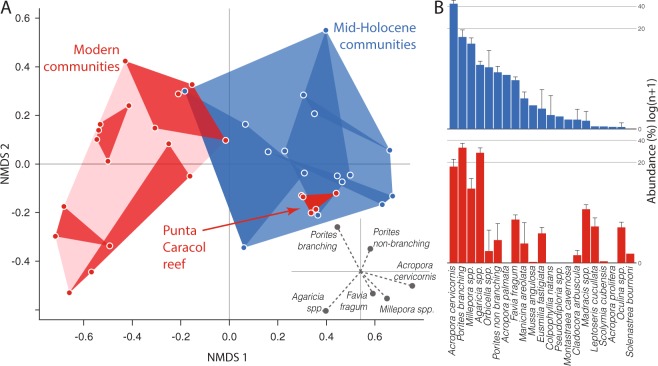
Most modern coral communities (red) are compositionally distinct from their fossil counterparts (blue) except the living reef at Punta Caracol. (**A**) Non-metric multidimensional scaling (NMDS) ordination place communities in multivariate ecological space based on the relative abundance of coral taxa skeletal weights^50^. Inset shows the vector and magnitude of the scores from the six most explanatory taxa. (**B**) Rank abundance plots (log-scale) show shifts in the dominant and rare taxa between mid-Holocene (top) and modern (bottom) reefs.

We used the relative abundances of coral taxa in the fossil samples to define the HRV of the reefs in the mid-Holocene and compare that HRV to the community composition of modern adjacent reefs. Ordination of the data demonstrates that, overall, the modern coral communities in Almirante Bay are distinct from the fossil-defined HRV (Fig. 3). The ecological space occupied by mid-Holocene coral communities, estimated by NMDS, is significantly different (PERMANOVA; p < 0.0001) from modern coral communities in the same area. Coral communities in this reef system today are therefore distinct from their mid-Holocene counterparts, suggesting that the modern reefs exist in novel states.

Mid-Holocene Caribbean reefs were generally dominated by staghorn coral (*Acropora cervicornis*), which was rare or absent on many of the modern reefs in our study (Fig. S4). This finding is consistent with other paleoecological work showing that acroporid corals were the principal pre-Anthropocene reef-builders in the Caribbean^64,65^ before historical^1^ and more recent^23,66^ declines in acroporid corals. Modern coral communities on the fringing reef we studied are instead primarily dominated by finger coral (branching *Porites* spp.) or lettuce leaf coral (*Agaricia* spp.) (Fig. 3), corroborating results from benthic surveys that show *Agaricia* and branching *Porites* have dominated these reefs, and others across Almirante Bay, for at least the last 20 years^32,56,67^. This trend also mirrors the Caribbean-wide transition from acroporid to *Agaricia* and branching *Porites* dominance^56,68,69^ frequently attributed to *Agaricia* and *Porites* being more robust to stress and disease than *Acropora*^28^. The drivers of this region-wide shift remain under debate with researchers recognising both a historical and modern component to the transition, as well as regional and local determinants^2,70^.

### A resilient reef at Punta Caracol

In contrast to the shift to novel ecosystem states observed in all of the other modern reefs sampled in this study, we found that the coral community composition at one modern reef – Punta Caracol – is entirely contained within the fossil-defined HRV (Fig. 3). Like the mid-Holocene reefs, coral assemblages at Punta Caracol are dominated by *A. cervicornis* (Fig. S4). Benthic surveys show that *A. cervicornis* has existed on the reef since at least 1997^67,71^. To reveal the deeper history of this reef, we collected two push-cores following methods described in Cramer *et al*.^20^. Cores were taken ~2 m apart from each other at a water depth of 2.2 m on top of living coral. In each core, five 5 cm-deep samples were extracted from the core matrix every 10 cm, washed and sieved to >2 mm and all coral pieces identified^50^. Results show that this reef was dominated by *Acropora cervicornis*, without apparent interruption, for over 800 years (Fig. S5), as estimated using U-Th dating (Table S1). When taken together, these findings suggest that Punta Caracol is a reef that has evaded the shift to a novel state and retains taxonomic and functional elements of the reefs before human impact. We do not claim that the reef at Punta Caracol is “pristine”. The reef could not have avoided the impacts of conch^72^, lobster and fish harvesting^46^, regional and global pollution^32^ and global change in water temperature and chemistry^34^. Nevertheless, in spite of these stressors, the coral community has evaded the region-wide shift to a novel state that has impacted other reefs in the area.

Determining why Punta Caracol is an exceptional reef that has resisted the shift to novel states could reveal useful information that can be repurposed for the conservation of other reefs. Surprisingly though, we see no clear differences in the reef’s abiotic characteristics (i.e., topography, water flow, water residence time, frequency of hypoxia, suspended solids, dissolved nutrients and temperature) or biotic characteristics (i.e., planktonic productivity, coral cover, fish abundance)^32,34,73,74^ when compared to other reefs in the region that could account for why Punta Caracol is exceptional. Measuring local anthropogenic stressors such as fishing pressure, pollution and tourism was outside the scope of this paper, but of all the modern reefs studied here, the Punta Caracol reef is located farthest from the densest human population in the region (Fig. 2) This implicates a lack of local human-disturbance as the reason for the reef’s resilience. Alternatively, Punta Caracol could be a fortunate ‘oasis’^75^, although the 760-year stability in community composition (Fig. S5) undermines such a stochastic explanation, which would likely have manifested in shifting coral community composition over time. Finally, feedback mechanisms, such as the positive density-dependent increase in resilience of dominant patches of *A. cervicornis*^76^ (up to certain densities^77^) might have played a role in maintaining community stability on the reef. *A. cervicornis*, like most acroporid corals, is a species that has traded disease and predation-resistance for fast-growth and high-rates of clonal reproduction^23^. Previous work has shown that the population of *A. cervicornis* at the Punta Caracol reef has particularly low genetic diversity relative to other conspecific populations in the Bocas del Toro region, a sign that it has been isolated from other populations and established principally through clonal propagation^71^. Could a resilient, genetically isolated population of *A. cervicornis* have persisted for more than 800 years at Punta Caracol through clonal propagation? We propose that historical information, such as the data presented here, can be used to help make more informed choices when selecting resilient genets to translocate coral colonies, improve connectivity and recruitment^78^ and promote recovery^79^.

### Past variation as a guide to future conservation

There is a growing trend in conservation biology to accept that the majority of ecosystems today have already shifted into irreversible novel states and, consequently, that conservation resources should not be squandered trying to coerce restoration to unachievable historical conditions. For example, the near-ecological extinction of megafauna and fish from overharvesting and the chronic and intensifying effects of successive coral bleaching make returning Caribbean coral reefs to their past states almost unimaginable. While generally true, this paradigm involves the assumption that historical conditions of Caribbean coral communities were universally of high coral cover and high fish biomass^80^, a notion that persists even though reefs exhibit considerable variation in these measures today^2^ and did so in the past^16^. This assumption is reinforced by space-for-time substitution studies that take reefs that are perceived to be healthy and presume their states are the baselines for reefs perceived to be degraded. Our study highlights the pitfalls of the use of space-for-time substitution approaches in coral reefs. Not even an experienced reef ecologist is likely to think that Punta Caracol resembles a pre-human impact Caribbean reef because living coral cover and fish biomass, often used as a comparative metric of reef health, are not particularly high on this reef ^32^. Yet despite failing to fulfil expectations of what many might consider coral reefs should look like in the absence of humans, we demonstrate here, through the use of the HRV approach, that the coral community at Punta Caracol has persisted for centuries in a state that retains at least some elements of their prior condition before human impact.

**Figure 4:**
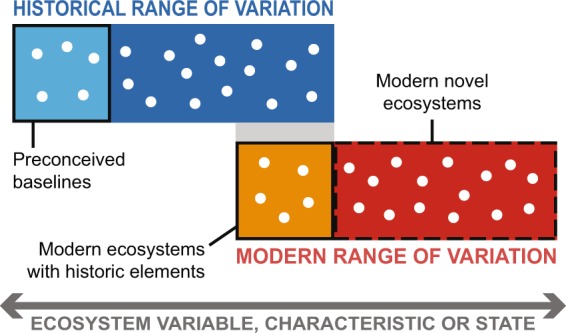
Quantifying the Historical Range of Variation (HRV) using fossils and comparing them to modern ecosystems can redefine preconceptions of pre-human ecosystems, establish which ecosystem states are truly novel, and reveal ecosystems that retain elements of their prior condition before human impact. Cartoon of localities (dots) along a gradient of ecosystem state, characteristic or other measurable ecological or environmental variable.

Defining the HRV of an ecosystem using the fossil record can therefore provide powerful, location-specific context for modern ecosystem change (Fig. 4), which can offer clues as to the ecosystem types, localities and genets that could confer functional, taxonomic and genetic elements useful for conservation. This could be valuable because it is difficult to predict how complex ecosystems like coral reefs will change in the future because of processes like extinction debt^81^, the unclear directionality of future ecosystem shifts^70^ and the complex impact of unidentified future stressors^82^. Conservationists interested in supporting coral reefs must therefore hedge their bets by conserving variability itself to provide nature with the raw material for the selection of resilience^83^. Resources for conservation will always be limited and decisions must be made about where priority should be given. We suggest that such decisions be made not only based on current states and future predictions but also through the use of HRV data to extend time scales, establish location-specific baselines and uncover elements of resilience that might otherwise be overlooked.

## Data availability

All data supporting the findings of this study are available within the paper and its Supporting Information files.

## Supplementary information

Supplementary Information.
