# Supplementary material for: Defining variation in pre-human ecosystems can guide conservation: An example from a Caribbean coral reef
Source: Sci Rep. 2020 Feb 19;10:2922. doi: 10.1038/s41598-020-59436-y (PMC7031243; doi:10.1038/s41598-020-59436-y)
Supplement: Supplementary file 1 — Supplementary Information. [file 41598_2020_59436_MOESM1_ESM.pdf]

Electronic Supplementary Material for

Defining variation in pre-human ecosystems can guide conservation: An example from a Caribbean coral reef

Aaron O'Dea[1], Mauro Lepore[1,2], Andrew H. Altieri[1,3], Melisa Chan[a], Jorge Manuel Morales-Saldaña[1], Nicte-Ha Muñoz[1], John M. Pandolfi[4], Marguerite A. Toscano[5], Jian-xin Zhao[6] and Erin M. Dillon[1,7]

[1] Smithsonian Tropical Research Institute, Box 0843-03092, Balboa, Republic of Panama.

[2] Smithsonian Institution, National Museum of Natural History, Washington, DC 20560-0121, USA.

[3] Department of Environmental Engineering Sciences, University of Florida, Gainesville, FL 32611, USA.

[4] School of Biological Sciences, University of Queensland, St. Lucia, Queensland 4072, Australia.

[5] Smithsonian Environmental Research Center, 647 Contees Wharf Road, Edgewater, Maryland 21037, USA.

[6] School of Earth & Environmental Sciences, University of Queensland, St. Lucia, Queensland 4072, Australia.

[7] Department of Ecology, Evolution and Marine Biology and the Marine Science Institute, University of California, Santa Barbara, CA 93106, USA

Corresponding author: Aaron O'Dea, odeaa@si.edu, +507 2128065

This file includes

Supplemental radiometric dating methods

figures S1 to S4

Tables S1 and S2

References for supplementary material

Supplemental radiometric dating methods

U–Th dating was conducted on coral fragments using a Nu Plasma multi-collector inductively-coupled plasma mass spectrometer (MC-ICP-MS) in the Radiogenic Isotope Facility at the University of Queensland, following chemical treatment procedures and MC-ICP-MS analytical protocols described in Clark et al. [1]. For U–Th dating, each coral sample consisting of ~150 mg fine sand-size chips that were carefully fragmented, H_2O_2-treated and then hand-picked under a binocular microscope to remove any trace detritus or grains with discolouration was spiked with a mixed ^{229}Th–^{233}U tracer and then completely dissolved in double-distilled concentrated HNO_3. After digestion, each sample was further treated with H_2O_2 to decompose trace amounts of organic matter (if any) and to facilitate complete sample-tracer homogenization. U and Th were separated using conventional anion-exchange column chemistry using Bio-Rad AG 1-X8 resin. After stripping off the matrix from the column using double-distilled 7N HNO_3 as eluent, $_4$N HNO_3 and 2%HNO_3 +0.03% HF mixture were used to elute U and Th into 3.5 ml pre-cleaned test tubes, respectively. After screening U and Th concentrations in the U and Th fractions using their 1:100 dilute solutions on a quadrupole ICP-MS, the U and Th separates for the samples were then re-mixed in 2% HNO_3 to make ~3 ml solution in a pre-cleaned 3.5 ml test tube. The mixed solution for each sample contained the entire Th fraction and a small percentage of the U fraction. The amount of U fraction to be added to the mixed solution was calculated based on the screening results and the MC-ICP-MS working sensitivity of the day, aiming to achieve ~5 volts of ^{238}U signal. The remixed U–Th solution was injected into the MC-ICP-MS through a DSN-100 desolvation nebulizer system with an uptake rate of ~0.06 ml/min. U–Th isotopic measurements were performed on the MC-ICP-MS using a detector configuration to allow simultaneous measurements of both U and Th isotopes. The $^{230}Th/^{238}U$ and $^{234}U/^{238}U$ activity ratios of the samples were calculated using the decay constants given in Cheng et al. [2]. ^{230}Th ages were calculated using the Isoplot 3.75 Program [3]. ^{230}Th ages were corrected for non-radiogenic ^{230}Th contributions using a modelled two-component-mixing non-radiogenic $^{230}Th/^{232}Th$ value based on the equation in Clark et al. [1].

We also obtained four AMS ^{14}C dates on exceptionally-well preserved branches of Acropora palmata (electronic supplementary material figure S4) from Trench 4 (figure 2). A. palmata generally occupies a narrow range of <1 to 5 (but can be up to 15) m water depth [4,5], but in the low-visibility waters of Almirante bay has only been observed in depths <2 m (A. Castillo, pers. comm.). Robust branches were found in the trenches at elevations of -3 to -1.5 m below mean modern sea level in some of the trenches (figure 2). Samples for dating were washed and air-dried, cut into slabs, and subsampled to retrieve pristine interior material without encrusters, epibionts and submarine cements. Prior to dating, X-Ray Diffraction was undertaken to determine if the samples consisted of 100 percent Aragonite; all samples were chemically pristine with no recrystallization or internal cements. Cleaned coral pieces were radiocarbon-dated at University of Georgia Center for Applied Isotope Studies. Coral pieces were first treated with dilute HCl to remove any contamination from the surface. The washed and dried sample was treated in the vacuum with concentrated phosphoric acid to recover carbon dioxide. For accelerator mass spectrometry analysis the cleaned samples were combusted at 900°C in evacuated / sealed ampoules in the presence of CuO. Resulting carbon dioxide was cryogenically purified from the other reaction products and catalytically converted to graphite using the method of Vogel et al. [6]. Graphite $^{14}C/^{13}C$ ratios were measured using the CAIS 0.5 MeV accelerator mass spectrometer. The sample ratios were compared to the ratio measured from the Oxalic Acid I (NBS SRM 4990). The sample $^{13}C/^{12}C$ ratios were measured separately using a stable isotope ratio mass spectrometer and expressed as $\delta^{13}C$ with respect to PDB, with an error of less than 0.1‰. The quoted uncalibrated dates have been given in radiocarbon years before 1950 (years BP), using the ^{14}C half-life of 5568 years, with error quoted as one standard deviation reflecting both statistical and experimental errors. The date has been corrected for isotope fractionation. Recovered dates were calibrated to calendar years before 1950 (cal BP) using CALIB 7.02 [7,8], the Marine13 calibration dataset [9], and an integrated, time-dependent global ocean reservoir correction. The age difference between the local ocean reservoir and modeled values (DR) was set at −36 ± 35 yr for the Gulf of Mexico [10]. One and two sigma calibrated ranges are reported with CALIB median probability values (electronic supplementary material, Table S1).

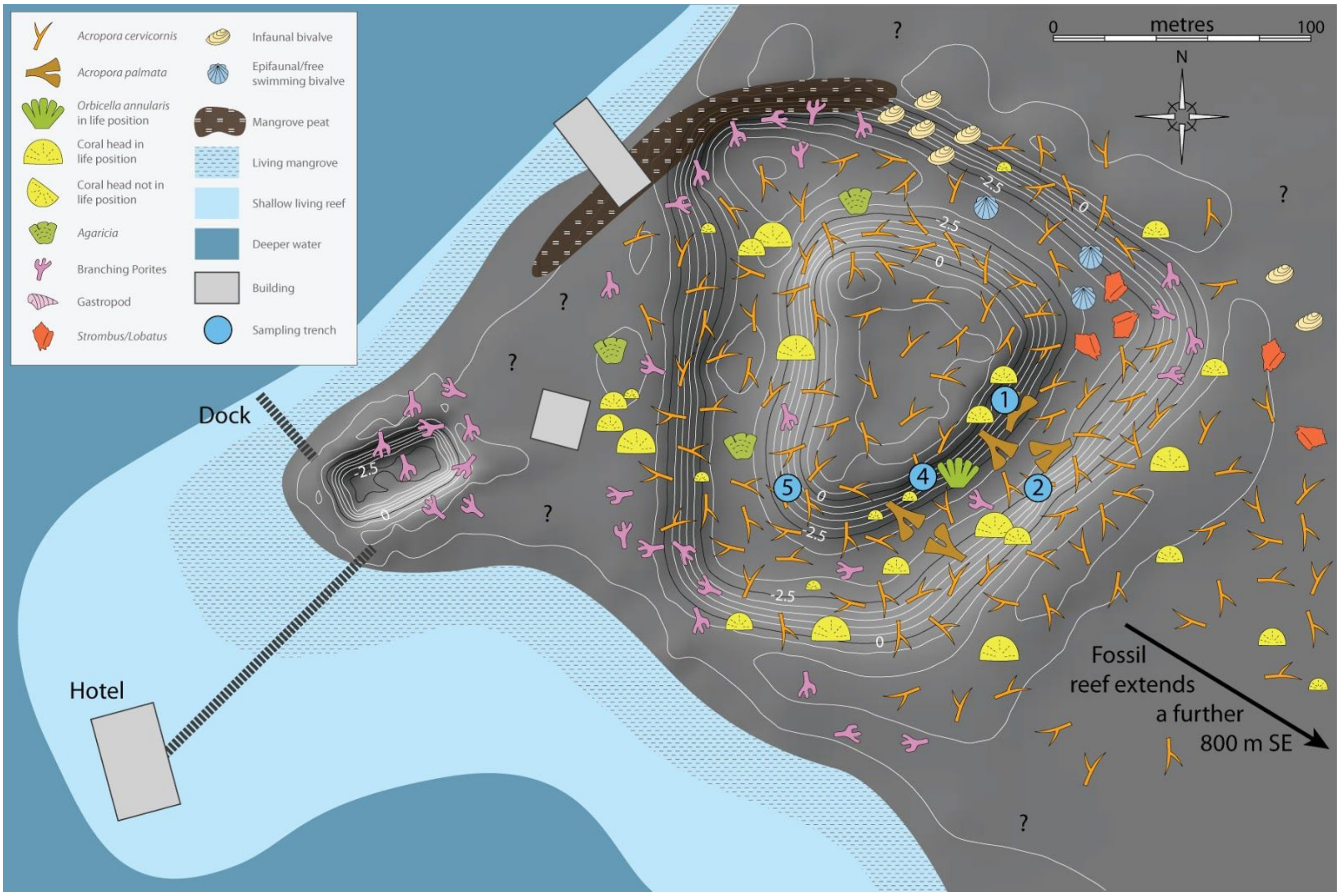

Figure S1. 3D model and habitat (facies) map of the NW portion of the mid-Holocene fossil reef site at Sweet Bocas, Bocas del Toro, Panama. Distribution of the remaining parts of the fossil reef are detailed in [11]. Contours in metres relative to MSL (see Materials and Methods for further information). The location of dominant fossils in the exposed surface matrix are labelled and the location of excavated trenches used in this study are marked. Map is centred on 9.359898°, -82.272677°. Drawn with Trimble Positions v10.1.0.3 https://www.trimble.com

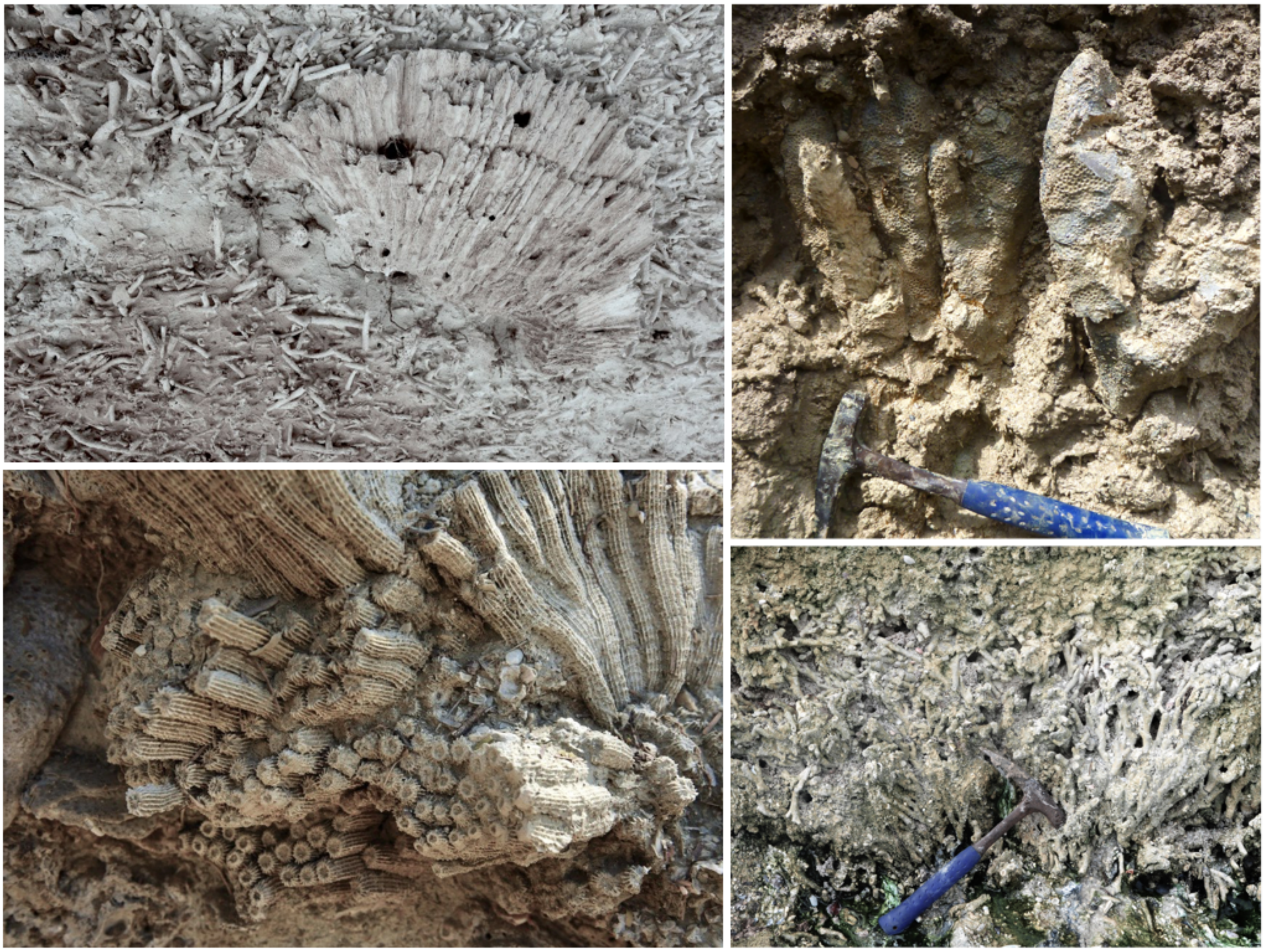

Figure S2. *In situ* and in life position corals exposed after trenching the mid-Holocene reef at Sweet Bocas, Bocas del Toro, Panamá**.** (a) a large coral head (possibly *Diploria*) in life position surrounded by fragments of *Acropora cervicornis*. (b) Columnar *Orbicella annularis* colony in life position. (c) Colony of *Montastraea cavernosa* in life position. (d) Large colony of branching *Porites sp.* in life position.

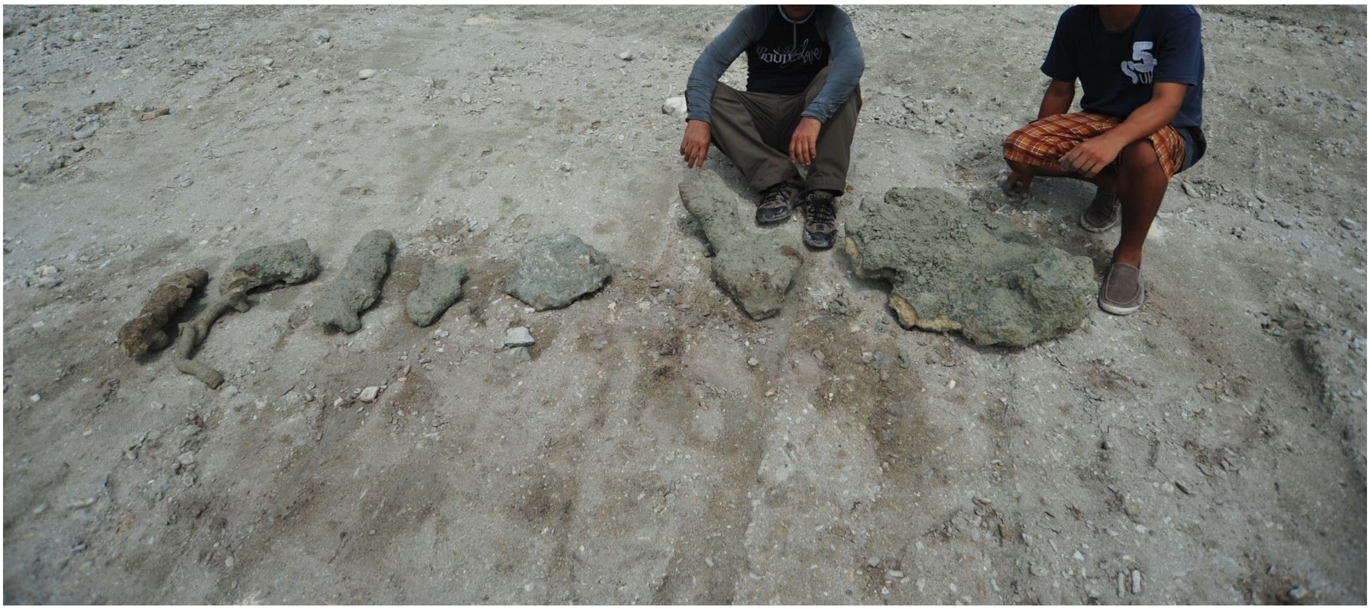

Figure S3. Examples of the many large fronds of well-preserved, chemically-pristine *Acropora palmata* colonies found in the mid-Holocene reef.

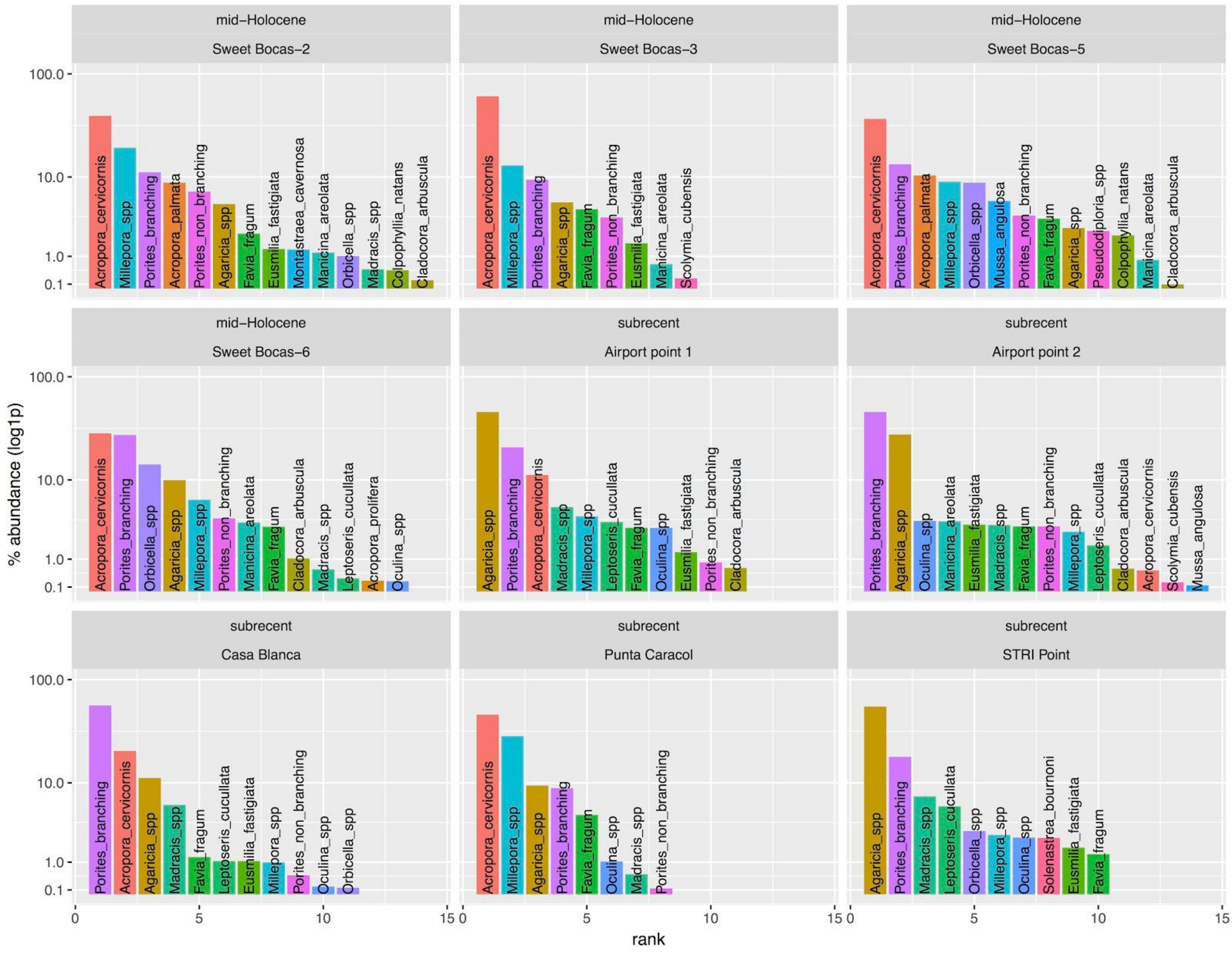

Figure S4. Rank-abundances (relative abundances based on mass of skeletal remains) of coral taxa in the modern reefs and mid-Holocene trenches.

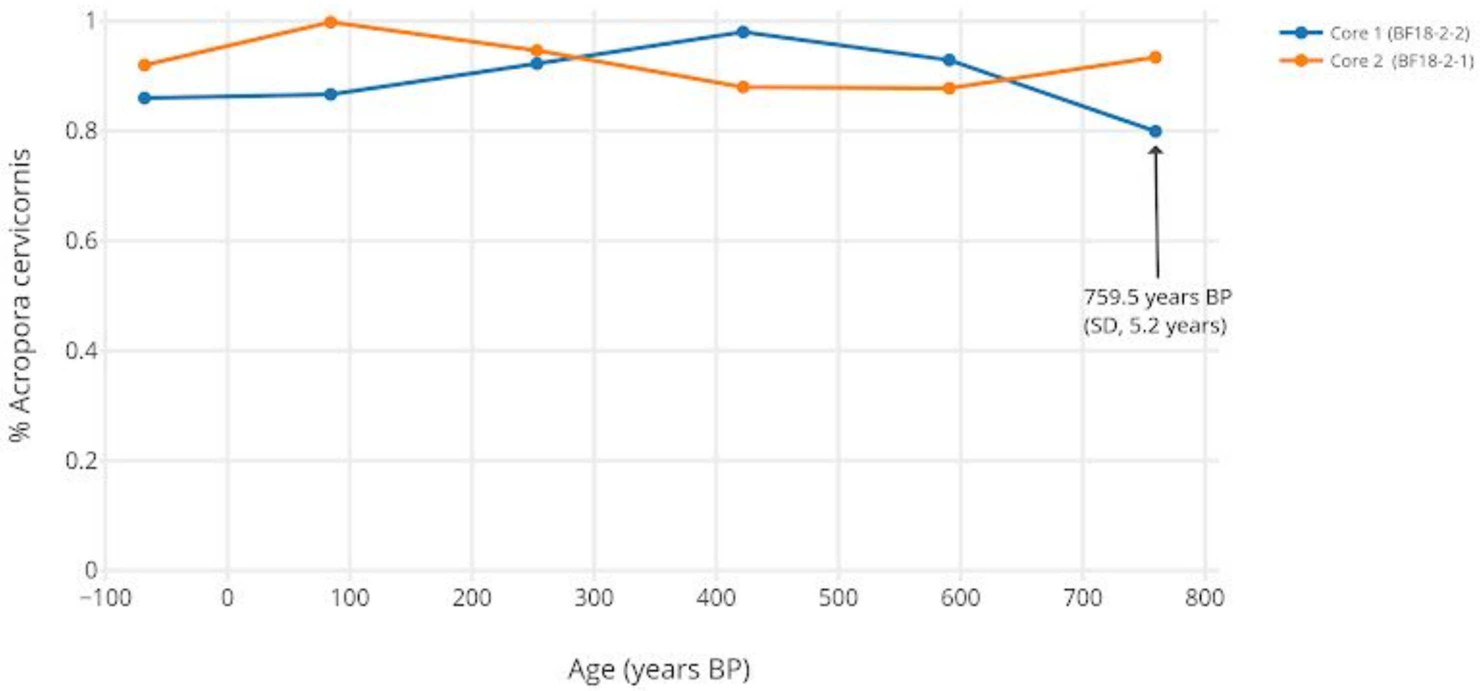

Figure S5. *Acropora cervicornis* dominated the reef at Punta Caracol over the last ~800 years. Relative abundance (proportion mass) of *A. cervicornis* to all other corals (including *Millepora*) in two cores taken ~2 m apart on the reef at Punta Caracol at 2.2 m water depth. Both cores included living coral at their tops. Chronology was estimated by dating a coral piece using U-Th (759.5 yBP) in the first core and assuming linear accretion rate to present day (2019) based on living coral at the top of the core.

Table S1. Radiometric dates used in the study. U-Th and calibrated-radiocarbon dating from coral pieces provide age control on modern and fossil samples to determine extent of time averaging (top), estimate reef accumulation rates at Punta Caracol reef (middle) and determine the age of the mid-Holocene reef (bottom). Almost all coral skeletons were chemically original with no calcite as observed from x-ray diffraction [11], and preservation generally exquisite with little bioerosion. * = mean taken from three measurements on same coral.

Sample number	Site	Type	Method	medium	Age (years BP)	Year AD	95% CI (years)	Ref.
To determine extent of time averaging in modern assemblages								
AT13-10-1-1	Punta Caracol	Bulk	U-Th	*Acropora cervicornis*	-58.1	2008	1.7	This study
AT13-10-2-1	Punta Caracol	Bulk	U-Th	*Acropora cervicornis*	-62.3	2012	1.2	This study
AT13-10-2-1	Punta Caracol	Bulk	U-Th	*Acropora cervicornis*	-58.2	2008	1.5	This study
AT13-10-3-1	Punta Caracol	Bulk	U-Th	*Acropora cervicornis*	-57.2	2007	1.4	This study
AT13-10-4-1	Punta Caracol	Bulk	U-Th	*Acropora cervicornis*	-52.5	2002	2.1	This study
AT13-10-5-1	Punta Caracol	Bulk	U-Th	*Acropora cervicornis*	-62.8	2012	1.3	This study
VC13-4-2-[0-5]	Cayo Adriana	Core top	U-Th	*Acropora cervicornis*	24	1926	NA	[12]
VC13-3-1-[0-5]	Punto Donato	Core top	U-Th	*Acropora cervicornis*	-34	1984	NA	[12]
VC13-3-1-[0-5]	Punto Donato	Core top	U-Th	*Acropora cervicornis*	-6	1956	NA	[12]
To estimate reef chronology at Punta Caracol								
BF18-2-2-[40-45]	Punta Caracol	Core	U-Th	*Acropora cervicornis*	759.5	1190	5.2	This study
To determine age of reefs in mid-Holocene site								
AFH12-1a	Sunset Point	Hand	U-Th	Coral head	7187		8	[11]
AFH12-1b	Sunset Point	Hand	U-Th	Coral head	6708		8	[11]
AFH12-1c	Sunset Point	Hand	U-Th	Coral head	6702*		42	[11]
AFH12-1d	Sunset Point	Hand	U-Th	Coral head	6671		3	[11]
AFH12-1e	Sunset Point	Hand	U-Th	Coral head	6021		3	[11]
AFH12-1f	Sunset Point	Hand	U-Th	Coral head	5834*		5	[11]
AFH12-1g	Sunset Point	Hand	U-Th	Coral head	5818		7	[11]
AFH12-1h	Sunset Point	Hand	U-Th	Coral head	5781*		12	[11]
AFH12-1i	Sunset Point	Hand	U-Th	Coral head	5711		3	[11]
AT13-2-8	Sweet Bocas	Bulk	U-Th	*Acropora cervicornis*	6719		13.3	This study
AT13-3-11	Sweet Bocas	Bulk	U-Th	*Acropora cervicornis*	6982		21.5	This study
AT13-51A	Sweet Bocas	Hand	^{14}C	*Acropora palmata*	6638		-	This study
AT13-51A	Sweet Bocas	Hand	^{14}C	*Acropora palmata*	6638		-	This study
AT13-52A	Sweet Bocas	Hand	^{14}C	*Acropora palmata*	6589		-	This study
AT13-52A	Sweet Bocas	Hand	^{14}C	*Acropora palmata*	6589		-	This study
AT13-53A	Sweet Bocas	Hand	^{14}C	*Acropora palmata*	6626		-	This study
AT13-53A	Sweet Bocas	Hand	^{14}C	*Acropora palmata*	6626		-	This study
AT13-56A	Sweet Bocas	Hand	^{14}C	*Acropora palmata*	6533		-	This study
AT13-56A	Sweet Bocas	Hand	^{14}C	*Acropora palmata*	6533		-	This study

Table S2. Data and locality information of samples used in this study. Raw data of coral taxa abundances (mass) in mid-Holocene and modern samples from Almirante Bay, Bocas del Toro.

observ_order	sample	Age	Reef	Long	Lat	Depth (m)	genus	species	mass_g
MC_16	AT12-4-3-2	Sub Recent	SW Bocas Island	-82.25418	9.33184	6	Porites	Porites spp_dfp	0.51
MC_16	AT12-4-3-2	Sub Recent	SW Bocas Island	-82.25418	9.33184	6	Unknown	unknown	41.51
MC_16	AT12-4-3-2	Sub Recent	SW Bocas Island	-82.25418	9.33184	6	Agaricia	Agaricia spp	51.05
MC_15	AT12-4-5-1	Sub Recent	SW Bocas Island	-82.25418	9.33184	10	Acropora	Acropora cervicornis	0.24
MC_15	AT12-4-5-1	Sub Recent	SW Bocas Island	-82.25418	9.33184	10	Madracis	Madracis spp	1.06
MC_15	AT12-4-5-1	Sub Recent	SW Bocas Island	-82.25418	9.33184	10	Oculina	Oculina spp	0.65
MC_15	AT12-4-5-1	Sub Recent	SW Bocas Island	-82.25418	9.33184	10	Porites	Porites spp_dfp	0.84
MC_15	AT12-4-5-1	Sub Recent	SW Bocas Island	-82.25418	9.33184	10	Favia	Favia fragum	1.59
MC_15	AT12-4-5-1	Sub Recent	SW Bocas Island	-82.25418	9.33184	10	Leptoseris	Leptoseris cucullata	2.19
MC_15	AT12-4-5-1	Sub Recent	SW Bocas Island	-82.25418	9.33184	10		other not coral	4.64
MC_15	AT12-4-5-1	Sub Recent	SW Bocas Island	-82.25418	9.33184	10	Agaricia	Agaricia spp	36.86
MC_15	AT12-4-5-1	Sub Recent	SW Bocas Island	-82.25418	9.33184	10	Unknown	unknown	44.93
ML_8	AT13-10-1-1	Sub Recent	Punta Caracol	-82.30383333	9.37845	1.9	Oculina	Oculina diffusa	0.14
ML_8	AT13-10-1-1	Sub Recent	Punta Caracol	-82.30383333	9.37845	1.9	Agaricia	Agaricia fragilis	0.66
ML_8	AT13-10-1-1	Sub Recent	Punta Caracol	-82.30383333	9.37845	1.9	Favia	Favia fragum	1.88
ML_9	AT13-10-2-1	Sub Recent	Punta Caracol	-82.30383333	9.37845	1.9	Porites	Porites colonensis	0.10
ML_8	AT13-10-1-1	Sub Recent	Punta Caracol	-82.30383333	9.37845	1.9	Porites	Porites spp_dfp	3.68
ML_8	AT13-10-1-1	Sub Recent	Punta Caracol	-82.30383333	9.37845	1.9	Agaricia	Agaricia agaricites	9.58
ML_8	AT13-10-1-1	Sub Recent	Punta Caracol	-82.30383333	9.37845	1.9	Millepora	Millepora spp	148.95
ML_8	AT13-10-1-1	Sub Recent	Punta Caracol	-82.30383333	9.37845	1.9	Acropora	Acropora cervicornis	228.69
ML_8	AT13-10-1-1	Sub Recent	Punta Caracol	-82.30383333	9.37845	1.9		other not coral	494.30
ML_9	AT13-10-2-1	Sub Recent	Punta Caracol	-82.30383333	9.37845	1.9	Agaricia	Agaricia humilis	9.31
ML_9	AT13-10-2-1	Sub Recent	Punta Caracol	-82.30383333	9.37845	1.9	Porites	Porites spp_dfp	4.10
ML_9	AT13-10-2-1	Sub Recent	Punta Caracol	-82.30383333	9.37845	1.9	Acropora	Acropora cervicornis	590.90
ML_9	AT13-10-2-1	Sub Recent	Punta Caracol	-82.30383333	9.37845	1.9		other not coral	412.10
ML_9	AT13-10-2-1	Sub Recent	Punta Caracol	-82.30383333	9.37845	1.9	Unknown	unknown	140.09
ML_9	AT13-10-2-1	Sub Recent	Punta Caracol	-82.30383333	9.37845	1.9	Millepora	Millepora spp	72.38
ML_9	AT13-10-2-1	Sub Recent	Punta Caracol	-82.30383333	9.37845	1.9	Agaricia	Agaricia lamarcki	1.72
ML_2	AT13-2-10-	Holocene	Sweet Bocas Trench 1	-82.27153333	9.360233333	URS	Porites	Porites divaricata	17.65
ML_9	AT13-10-2-1	Sub Recent	Punta Caracol	-82.30383333	9.37845	1.9	Agaricia	Agaricia agaricites	0.63
ML_10	AT13-10-3-1	Sub Recent	Punta Caracol	-82.30383333	9.37845	1.8	Porites	Porites spp_dfp	0.18
ML_9	AT13-10-2-1	Sub Recent	Punta Caracol	-82.30383333	9.37845	1.9	Madracis	Madracis spp	0.19
ML_10	AT13-10-3-1	Sub Recent	Punta Caracol	-82.30383333	9.37845	1.8	Acropora	Acropora cervicornis	259.74
NM_5	AT13-10-4-1	Sub Recent	Punta Caracol	-82.30383333	9.37845	1.8	Agaricia	Agaricia tenuifolia	0.23
NM_5	AT13-10-4-1	Sub Recent	Punta Caracol	-82.30383333	9.37845	1.8	Favia	Favia fragum	5.17
ML_10	AT13-10-3-1	Sub Recent	Punta Caracol	-82.30383333	9.37845	1.8		other not coral	1008.70
NM_5	AT13-10-4-1	Sub Recent	Punta Caracol	-82.30383333	9.37845	1.8	Agaricia	Agaricia lamarcki	0.22
NM_5	AT13-10-4-1	Sub Recent	Punta Caracol	-82.30383333	9.37845	1.8	Agaricia	Agaricia agaricites	3.21
NM_5	AT13-10-4-1	Sub Recent	Punta Caracol	-82.30383333	9.37845	1.8	Porites	Porites spp_dfp	4.46
NM_5	AT13-10-4-1	Sub Recent	Punta Caracol	-82.30383333	9.37845	1.8	Agaricia	Agaricia fragilis	1.39
ML_10	AT13-10-3-1	Sub Recent	Punta Caracol	-82.30383333	9.37845	1.8	Agaricia	Agaricia agaricites	1.60
ML_10	AT13-10-3-1	Sub Recent	Punta Caracol	-82.30383333	9.37845	1.8	Madracis	Madracis decactis	0.42
ML_10	AT13-10-3-1	Sub Recent	Punta Caracol	-82.30383333	9.37845	1.8	Agaricia	Agaricia spp	1.75
ML_10	AT13-10-3-1	Sub Recent	Punta Caracol	-82.30383333	9.37845	1.8	Agaricia	Agaricia lamarcki	3.42
ML_2	AT13-2-10-	Holocene	Sweet Bocas Trench 1	-82.27153333	9.360233333	URS	Unknown	unknown	45.30
ML_10	AT13-10-3-1	Sub Recent	Punta Caracol	-82.30383333	9.37845	1.8	Millepora	Millepora spp	224.45
ML_10	AT13-10-3-1	Sub Recent	Punta Caracol	-82.30383333	9.37845	1.8	Agaricia	Agaricia humilis	2.84
ML_10	AT13-10-3-1	Sub Recent	Punta Caracol	-82.30383333	9.37845	1.8	Unknown	unknown	218.53
ML_10	AT13-10-3-1	Sub Recent	Punta Caracol	-82.30383333	9.37845	1.8	Oculina	Oculina diffusa	0.40
ML_10	AT13-10-3-1	Sub Recent	Punta Caracol	-82.30383333	9.37845	1.8	Favia	Favia fragum	2.53
ML_9	AT13-10-2-1	Sub Recent	Punta Caracol	-82.30383333	9.37845	1.9	Favia	Favia fragum	6.65
ML_8	AT13-10-1-1	Sub Recent	Punta Caracol	-82.30383333	9.37845	1.9	Unknown	unknown	97.79

NM_5	AT13-10-4-1	Sub Recent	Punta Caracol	-82.30383333	9.37845	1.8	Agaricia	Agaricia humilis	14.13
NM_5	AT13-10-4-1	Sub Recent	Punta Caracol	-82.30383333	9.37845	1.8	Unknown	unknown	233.15
NM_6	AT13-10-5-1	Sub Recent	Punta Caracol	-82.30383333	9.37845	1.7	Agaricia	Agaricia agaricites	4.95
NM_6	AT13-10-5-1	Sub Recent	Punta Caracol	-82.30383333	9.37845	1.7	Agaricia	Agaricia undata	1.23
NM_5	AT13-10-4-1	Sub Recent	Punta Caracol	-82.30383333	9.37845	1.8		other not coral	309.86
NM_6	AT13-10-5-1	Sub Recent	Punta Caracol	-82.30383333	9.37845	1.7	Agaricia	Agaricia lamarcki	1.60
NM_6	AT13-10-5-1	Sub Recent	Punta Caracol	-82.30383333	9.37845	1.7	Agaricia	Agaricia humilis	3.17
NM_5	AT13-10-4-1	Sub Recent	Punta Caracol	-82.30383333	9.37845	1.8	Millepora	Millepora spp	282.71
NM_5	AT13-10-4-1	Sub Recent	Punta Caracol	-82.30383333	9.37845	1.8	Agaricia	Agaricia spp	42.00
NM_6	AT13-10-5-1	Sub Recent	Punta Caracol	-82.30383333	9.37845	1.7	Oculina	Oculina spp	1.76
NM_5	AT13-10-4-1	Sub Recent	Punta Caracol	-82.30383333	9.37845	1.8	Acropora	Acropora cervicornis	513.83
NM_6	AT13-10-5-1	Sub Recent	Punta Caracol	-82.30383333	9.37845	1.7	Porites	Porites spp_dfp	44.44
NM_6	AT13-10-5-1	Sub Recent	Punta Caracol	-82.30383333	9.37845	1.7	Millepora	Millepora spp	176.36
NM_6	AT13-10-5-1	Sub Recent	Punta Caracol	-82.30383333	9.37845	1.7	Unknown	unknown	152.04
NM_6	AT13-10-5-1	Sub Recent	Punta Caracol	-82.30383333	9.37845	1.7	Acropora	Acropora cervicornis	393.87
ML_1	AT13-2-1-	Holocene	Sweet Bocas Trench 1	-82.27153333	9.360233333	URS	Madracis	Madracis decactis	4.49
NM_6	AT13-10-5-1	Sub Recent	Punta Caracol	-82.30383333	9.37845	1.7	Favia	Favia fragum	5.68
NM_6	AT13-10-5-1	Sub Recent	Punta Caracol	-82.30383333	9.37845	1.7		other not coral	582.93
ML_1	AT13-2-1-	Holocene	Sweet Bocas Trench 1	-82.27153333	9.360233333	URS	Agaricia	Agaricia agaricites	0.38
ML_1	AT13-2-1-	Holocene	Sweet Bocas Trench 1	-82.27153333	9.360233333	URS	Acropora	Acropora palmata	1153.50
ML_1	AT13-2-1-	Holocene	Sweet Bocas Trench 1	-82.27153333	9.360233333	URS	Porites	Porites colonensis	134.79
ML_1	AT13-2-1-	Holocene	Sweet Bocas Trench 1	-82.27153333	9.360233333	URS	Montastraea	Montastraea cavernosa	29.49
ML_1	AT13-2-1-	Holocene	Sweet Bocas Trench 1	-82.27153333	9.360233333	URS	Manicina	Manicina areolata	9.58
ML_1	AT13-2-1-	Holocene	Sweet Bocas Trench 1	-82.27153333	9.360233333	URS	cup	cup coral	0.34
ML_1	AT13-2-1-	Holocene	Sweet Bocas Trench 1	-82.27153333	9.360233333	URS	Porites	Porites furcata	24.27
ML_1	AT13-2-1-	Holocene	Sweet Bocas Trench 1	-82.27153333	9.360233333	URS	Porites	Porites divaricata	3.90
ML_1	AT13-2-1-	Holocene	Sweet Bocas Trench 1	-82.27153333	9.360233333	URS	Favia	Favia fragum	5.51
ML_1	AT13-2-1-	Holocene	Sweet Bocas Trench 1	-82.27153333	9.360233333	URS	Agaricia	Agaricia lamarcki	13.59
ML_1	AT13-2-1-	Holocene	Sweet Bocas Trench 1	-82.27153333	9.360233333	URS	Eusmilia	Eusmilia fastigiata	30.59
ML_1	AT13-2-1-	Holocene	Sweet Bocas Trench 1	-82.27153333	9.360233333	URS		other not coral	388.17
ML_1	AT13-2-1-	Holocene	Sweet Bocas Trench 1	-82.27153333	9.360233333	URS	Unknown	unknown	527.80
ML_1	AT13-2-1-	Holocene	Sweet Bocas Trench 1	-82.27153333	9.360233333	URS	Acropora	Acropora cervicornis	653.50
ML_2	AT13-2-10-	Holocene	Sweet Bocas Trench 1	-82.27153333	9.360233333	URS	Agaricia	Agaricia fragilis	12.77
ML_2	AT13-2-10-	Holocene	Sweet Bocas Trench 1	-82.27153333	9.360233333	URS	Cladocora	Cladocora arbuscula	0.21
ML_2	AT13-2-10-	Holocene	Sweet Bocas Trench 1	-82.27153333	9.360233333	URS	Porites	Porites spp_dfp	11.01
ML_2	AT13-2-10-	Holocene	Sweet Bocas Trench 1	-82.27153333	9.360233333	URS	Agaricia	Agaricia agaricites	11.19
ML_2	AT13-2-10-	Holocene	Sweet Bocas Trench 1	-82.27153333	9.360233333	URS	Millepora	Millepora spp	488.11
ML_2	AT13-2-10-	Holocene	Sweet Bocas Trench 1	-82.27153333	9.360233333	URS	Acropora	Acropora cervicornis	629.20
ML_3	AT13-2-8-	Holocene	Sweet Bocas Trench 1	-82.27153333	9.360233333	URS	Agaricia	Agaricia humilis	0.30
ML_3	AT13-2-8-	Holocene	Sweet Bocas Trench 1	-82.27153333	9.360233333	URS	Agaricia	Agaricia lamarcki	1.42
ML_3	AT13-2-8-	Holocene	Sweet Bocas Trench 1	-82.27153333	9.360233333	URS	Montastraea	Montastraea annularis	9.09
ML_3	AT13-2-8-	Holocene	Sweet Bocas Trench 1	-82.27153333	9.360233333	URS	Porites	Porites divaricata	1.55
ML_3	AT13-2-8-	Holocene	Sweet Bocas Trench 1	-82.27153333	9.360233333	URS	Porites	Porites furcata	14.55
ML_3	AT13-2-8-	Holocene	Sweet Bocas Trench 1	-82.27153333	9.360233333	URS	Porites	Porites astreoides	15.38
ML_3	AT13-2-8-	Holocene	Sweet Bocas Trench 1	-82.27153333	9.360233333	URS	Favia	Favia fragum	16.63
ML_3	AT13-2-8-	Holocene	Sweet Bocas Trench 1	-82.27153333	9.360233333	URS	Acropora	Acropora cervicornis	2006.60
ML_3	AT13-2-8-	Holocene	Sweet Bocas Trench 1	-82.27153333	9.360233333	URS	Manicina	Manicina areolata	1.56
ML_3	AT13-2-8-	Holocene	Sweet Bocas Trench 1	-82.27153333	9.360233333	URS		other not coral	4.32
ML_3	AT13-2-8-	Holocene	Sweet Bocas Trench 1	-82.27153333	9.360233333	URS	Millepora	Millepora spp	71.46
ML_3	AT13-2-8-	Holocene	Sweet Bocas Trench 1	-82.27153333	9.360233333	URS	Unknown	unknown	313.62
ML_4	AT13-2-9-	Holocene	Sweet Bocas Trench 1	-82.27153333	9.360233333	URS	Favia	Favia fragum	0.87
ML_4	AT13-2-9-	Holocene	Sweet Bocas Trench 1	-82.27153333	9.360233333	URS	Agaricia	Agaricia lamarcki	11.44
ML_4	AT13-2-9-	Holocene	Sweet Bocas Trench 1	-82.27153333	9.360233333	URS	Porites	Porites divaricata	15.50
ML_4	AT13-2-9-	Holocene	Sweet Bocas Trench 1	-82.27153333	9.360233333	URS	Agaricia	Agaricia agaricites	17.26
ML_4	AT13-2-9-	Holocene	Sweet Bocas Trench 1	-82.27153333	9.360233333	URS	Porites	Porites colonensis	118.17
ML_4	AT13-2-9-	Holocene	Sweet Bocas Trench 1	-82.27153333	9.360233333	URS	Porites	Porites spp_dfp	147.40
ML_4	AT13-2-9-	Holocene	Sweet Bocas Trench 1	-82.27153333	9.360233333	URS	Unknown	unknown	416.54

ML_4	AT13-2-9-	Holocene	Sweet Bocas Trench 1	-82.27153333	9.360233333	URS	Millepora	Millepora spp	620.30
ML_4	AT13-2-9-	Holocene	Sweet Bocas Trench 1	-82.27153333	9.360233333	URS	Acropora	Acropora cervicornis	638.85
ML_5	AT13-3-10-	Holocene	Sweet Bocas Trench 2	-82.27143333	9.359933333	URS	Agaricia	Agaricia tenuifolia	0.28
ML_5	AT13-3-10-	Holocene	Sweet Bocas Trench 2	-82.27143333	9.359933333	URS	Agaricia	Agaricia humilis	14.07
ML_5	AT13-3-10-	Holocene	Sweet Bocas Trench 2	-82.27143333	9.359933333	URS	Porites	Porites colonensis	12.84
ML_5	AT13-3-10-	Holocene	Sweet Bocas Trench 2	-82.27143333	9.359933333	URS	Porites	Porites divaricata	20.82
ML_5	AT13-3-10-	Holocene	Sweet Bocas Trench 2	-82.27143333	9.359933333	URS		other not coral	21.90
ML_5	AT13-3-10-	Holocene	Sweet Bocas Trench 2	-82.27143333	9.359933333	URS	Favia	Favia fragum	25.54
ML_5	AT13-3-10-	Holocene	Sweet Bocas Trench 2	-82.27143333	9.359933333	URS	Millepora	Millepora spp	76.72
MC_4	AT12-1-1-1	Sub Recent	STRI Point	-82.262555	9.349446	12	Madracis	Madracis spp	0.12
MC_4	AT12-1-1-1	Sub Recent	STRI Point	-82.262555	9.349446	12	Oculina	Oculina diffusa	0.14
MC_4	AT12-1-1-1	Sub Recent	STRI Point	-82.262555	9.349446	12	Favia	Favia fragum	0.24
MC_4	AT12-1-1-1	Sub Recent	STRI Point	-82.262555	9.349446	12	Eusmilia	Eusmilia fastigiata	0.38
MC_4	AT12-1-1-1	Sub Recent	STRI Point	-82.262555	9.349446	12	Solenastrea	Solenastrea bournoni	0.71
MC_4	AT12-1-1-1	Sub Recent	STRI Point	-82.262555	9.349446	12	Leptoseris	Leptoseris cucullata	0.72
MC_4	AT12-1-1-1	Sub Recent	STRI Point	-82.262555	9.349446	12	Millepora	Millepora spp	0.85
MC_4	AT12-1-1-1	Sub Recent	STRI Point	-82.262555	9.349446	12	Porites	Porites spp_dfp	2.72
MC_4	AT12-1-1-1	Sub Recent	STRI Point	-82.262555	9.349446	12		other not coral	27.28
MC_4	AT12-1-1-1	Sub Recent	STRI Point	-82.262555	9.349446	12	Agaricia	Agaricia spp	33.62
MC_4	AT12-1-1-1	Sub Recent	STRI Point	-82.262555	9.349446	12	Unknown	unknown	69.19
NM_15	AT12-1-2-1	Sub Recent	STRI Point	-82.262555	9.349446	10	Madracis	Madracis asperula	1.89
NM_15	AT12-1-2-1	Sub Recent	STRI Point	-82.262555	9.349446	10	Leptoseris	Leptoseris cucullata	0.34
NM_15	AT12-1-2-1	Sub Recent	STRI Point	-82.262555	9.349446	10	Orbicella	Orbicella spp	0.36
NM_15	AT12-1-2-1	Sub Recent	STRI Point	-82.262555	9.349446	10	Oculina	Oculina spp	0.45
NM_15	AT12-1-2-1	Sub Recent	STRI Point	-82.262555	9.349446	10	Madracis	Madracis decactis	1.35
NM_15	AT12-1-2-1	Sub Recent	STRI Point	-82.262555	9.349446	10		other not coral	2.14
NM_15	AT12-1-2-1	Sub Recent	STRI Point	-82.262555	9.349446	10	Porites	Porites spp_dfp	6.03
NM_15	AT12-1-2-1	Sub Recent	STRI Point	-82.262555	9.349446	10	Unknown	unknown	46.89
NM_15	AT12-1-2-1	Sub Recent	STRI Point	-82.262555	9.349446	10	Agaricia	Agaricia spp	112.15
MC_13	AT12-1-3-2	Sub Recent	STRI Point	-82.262555	9.349446	8		other not coral	0.67
MC_13	AT12-1-3-2	Sub Recent	STRI Point	-82.262555	9.349446	8	Orbicella	Orbicella spp	0.94
MC_13	AT12-1-3-2	Sub Recent	STRI Point	-82.262555	9.349446	8	Leptoseris	Leptoseris cucullata	1.42
MC_13	AT12-1-3-2	Sub Recent	STRI Point	-82.262555	9.349446	8	Madracis	Madracis decactis	2.29
MC_13	AT12-1-3-2	Sub Recent	STRI Point	-82.262555	9.349446	8	unknown	unknown	10.16
MC_13	AT12-1-3-2	Sub Recent	STRI Point	-82.262555	9.349446	8	Porites	Porites spp_dfp	24.71
MC_13	AT12-1-3-2	Sub Recent	STRI Point	-82.262555	9.349446	8	Agaricia	Agaricia spp	108.13
MC_12	AT12-4-1-2	Sub Recent	SW Bocas Island	-82.25418	9.33184	2	cup	cup coral	0.13
MC_12	AT12-4-1-2	Sub Recent	SW Bocas Island	-82.25418	9.33184	2	Cladocora	Cladocora arbuscula	0.20
MC_12	AT12-4-1-2	Sub Recent	SW Bocas Island	-82.25418	9.33184	2		other not coral	2.51
MC_12	AT12-4-1-2	Sub Recent	SW Bocas Island	-82.25418	9.33184	2	Millepora	Millepora spp	6.86
MC_12	AT12-4-1-2	Sub Recent	SW Bocas Island	-82.25418	9.33184	2	Acropora	Acropora cervicornis	11.86
MC_12	AT12-4-1-2	Sub Recent	SW Bocas Island	-82.25418	9.33184	2	Agaricia	Agaricia spp	40.51
MC_17	AT12-4-2-1	Sub Recent	SW Bocas Island	-82.25418	9.33184	4	Oculina	Oculina spp	3.93
MC_17	AT12-4-2-1	Sub Recent	SW Bocas Island	-82.25418	9.33184	4	Eusmilia	Eusmilia fastigiata	4.51
MC_17	AT12-4-2-1	Sub Recent	SW Bocas Island	-82.25418	9.33184	4	Millepora	Millepora spp	4.53
MC_17	AT12-4-2-1	Sub Recent	SW Bocas Island	-82.25418	9.33184	4	Madracis	Madracis spp	13.86
MC_17	AT12-4-2-1	Sub Recent	SW Bocas Island	-82.25418	9.33184	4	Porites	Porites spp_dfp	76.82
MC_17	AT12-4-2-1	Sub Recent	SW Bocas Island	-82.25418	9.33184	4	Acropora	Acropora cervicornis	80.81
MC_17	AT12-4-2-1	Sub Recent	SW Bocas Island	-82.25418	9.33184	4	Agaricia	Agaricia spp	89.00
MC_17	AT12-4-2-1	Sub Recent	SW Bocas Island	-82.25418	9.33184	4	Unknown	unknown	279.71
MC_16	AT12-4-3-2	Sub Recent	SW Bocas Island	-82.25418	9.33184	6	Madracis	Madracis spp	0.05
MC_16	AT12-4-3-2	Sub Recent	SW Bocas Island	-82.25418	9.33184	6	Acropora	Acropora cervicornis	0.09
ML_5	AT13-3-10-	Holocene	Sweet Bocas Trench 2	-82.27143333	9.359933333	URS	Unknown	unknown	410.97
ML_5	AT13-3-10-	Holocene	Sweet Bocas Trench 2	-82.27143333	9.359933333	URS	Acropora	Acropora cervicornis	1395.57
ML_6	AT13-3-11-	Holocene	Sweet Bocas Trench 2	-82.27143333	9.359933333	URS	Agaricia	Agaricia grahamae	24.29
ML_6	AT13-3-11-	Holocene	Sweet Bocas Trench 2	-82.27143333	9.359933333	URS	Porites	Porites astreoides	24.75
ML_6	AT13-3-11-	Holocene	Sweet Bocas Trench 2	-82.27143333	9.359933333	URS	Acropora	Acropora cervicornis	2128.77

ML_6	AT13-3-11-	Holocene	Sweet Bocas Trench 2	-82.27143333	9.359933333	URS	Scolymia	Scolymia cubensis	1.03
MC_12	AT12-4-1-2	Sub Recent	SW Bocas Island	-82.25418	9.33184	2	Porites	Porites spp_dfp	91.66
MC_12	AT12-4-1-2	Sub Recent	SW Bocas Island	-82.25418	9.33184	2	Unknown	unknown	96.41
MC_17	AT12-4-2-1	Sub Recent	SW Bocas Island	-82.25418	9.33184	4	Cladocora	Cladocora arbuscula	0.10
MC_17	AT12-4-2-1	Sub Recent	SW Bocas Island	-82.25418	9.33184	4		other not coral	0.16
MC_17	AT12-4-2-1	Sub Recent	SW Bocas Island	-82.25418	9.33184	4	Favia	Favia fragum	0.25
MC_17	AT12-4-2-1	Sub Recent	SW Bocas Island	-82.25418	9.33184	4	Leptoseris	Leptoseris cucullata	0.37
MC_17	AT12-4-2-1	Sub Recent	SW Bocas Island	-82.25418	9.33184	4	Porites	Porites astreoides	1.92
ML_6	AT13-3-11-	Holocene	Sweet Bocas Trench 2	-82.27143333	9.359933333	URS	Porites	Porites divaricata	3.57
ML_6	AT13-3-11-	Holocene	Sweet Bocas Trench 2	-82.27143333	9.359933333	URS		other not coral	7.40
ML_6	AT13-3-11-	Holocene	Sweet Bocas Trench 2	-82.27143333	9.359933333	URS	Favia	Favia fragum	9.09
ML_6	AT13-3-11-	Holocene	Sweet Bocas Trench 2	-82.27143333	9.359933333	URS	Porites	Porites spp_dfp	11.74
ML_6	AT13-3-11-	Holocene	Sweet Bocas Trench 2	-82.27143333	9.359933333	URS	Agaricia	Agaricia humilis	13.24
ML_6	AT13-3-11-	Holocene	Sweet Bocas Trench 2	-82.27143333	9.359933333	URS	Eusmilia	Eusmilia fastigiata	47.18
ML_6	AT13-3-11-	Holocene	Sweet Bocas Trench 2	-82.27143333	9.359933333	URS	Millepora	Millepora spp	83.81
ML_6	AT13-3-11-	Holocene	Sweet Bocas Trench 2	-82.27143333	9.359933333	URS	Unknown	unknown	258.49
ML_7	AT13-3-12-	Holocene	Sweet Bocas Trench 2	-82.27143333	9.359933333	URS	Porites	Porites astreoides	0.40
ML_7	AT13-3-12-	Holocene	Sweet Bocas Trench 2	-82.27143333	9.359933333	URS	Agaricia	Agaricia lamarcki	2.06
ML_7	AT13-3-12-	Holocene	Sweet Bocas Trench 2	-82.27143333	9.359933333	URS		other not coral	2.07
ML_7	AT13-3-12-	Holocene	Sweet Bocas Trench 2	-82.27143333	9.359933333	URS	Favia	Favia fragum	6.21
ML_7	AT13-3-12-	Holocene	Sweet Bocas Trench 2	-82.27143333	9.359933333	URS	Agaricia	Agaricia humilis	8.63
ML_7	AT13-3-12-	Holocene	Sweet Bocas Trench 2	-82.27143333	9.359933333	URS	Millepora	Millepora spp	59.24
ML_7	AT13-3-12-	Holocene	Sweet Bocas Trench 2	-82.27143333	9.359933333	URS	Unknown	unknown	282.66
ML_7	AT13-3-12-	Holocene	Sweet Bocas Trench 2	-82.27143333	9.359933333	URS	Acropora	Acropora cervicornis	2093.71
NM_1	AT13-3-13-	Holocene	Sweet Bocas Trench 2	-82.27143333	9.359933333	URS	Agaricia	Agaricia lamarcki	0.59
NM_1	AT13-3-13-	Holocene	Sweet Bocas Trench 2	-82.27143333	9.359933333	URS	Porites	Porites colonensis	0.74
NM_1	AT13-3-13-	Holocene	Sweet Bocas Trench 2	-82.27143333	9.359933333	URS	Porites	Porites divaricata	1.06
NM_1	AT13-3-13-	Holocene	Sweet Bocas Trench 2	-82.27143333	9.359933333	URS	Porites	Porites astreoides	1.12
NM_1	AT13-3-13-	Holocene	Sweet Bocas Trench 2	-82.27143333	9.359933333	URS	Agaricia	Agaricia agaricites	1.97
NM_1	AT13-3-13-	Holocene	Sweet Bocas Trench 2	-82.27143333	9.359933333	URS	Manicina	Manicina areolata	5.52
NM_1	AT13-3-13-	Holocene	Sweet Bocas Trench 2	-82.27143333	9.359933333	URS	Favia	Favia fragum	8.58
NM_1	AT13-3-13-	Holocene	Sweet Bocas Trench 2	-82.27143333	9.359933333	URS		other not coral	20.84
NM_1	AT13-3-13-	Holocene	Sweet Bocas Trench 2	-82.27143333	9.359933333	URS	Millepora	Millepora spp	27.54
NM_1	AT13-3-13-	Holocene	Sweet Bocas Trench 2	-82.27143333	9.359933333	URS	Unknown	unknown	376.92
NM_1	AT13-3-13-	Holocene	Sweet Bocas Trench 2	-82.27143333	9.359933333	URS	Acropora	Acropora cervicornis	2722.64
NM_2	AT13-3-14-	Holocene	Sweet Bocas Trench 2	-82.27143333	9.359933333	URS	Agaricia	Agaricia tenuifolia	0.12
NM_2	AT13-3-14-	Holocene	Sweet Bocas Trench 2	-82.27143333	9.359933333	URS	Agaricia	Agaricia spp	1.31
NM_2	AT13-3-14-	Holocene	Sweet Bocas Trench 2	-82.27143333	9.359933333	URS	Porites	Porites colonensis	1.43
NM_2	AT13-3-14-	Holocene	Sweet Bocas Trench 2	-82.27143333	9.359933333	URS	Favia	Favia fragum	1.89
NM_2	AT13-3-14-	Holocene	Sweet Bocas Trench 2	-82.27143333	9.359933333	URS		other not coral	2.40
NM_2	AT13-3-14-	Holocene	Sweet Bocas Trench 2	-82.27143333	9.359933333	URS	Porites	Porites astreoides	8.03
NM_2	AT13-3-14-	Holocene	Sweet Bocas Trench 2	-82.27143333	9.359933333	URS	Agaricia	Agaricia humilis	9.81
NM_2	AT13-3-14-	Holocene	Sweet Bocas Trench 2	-82.27143333	9.359933333	URS	Agaricia	Agaricia agaricites	10.50
NM_2	AT13-3-14-	Holocene	Sweet Bocas Trench 2	-82.27143333	9.359933333	URS	Porites	Porites furcata	36.33
NM_2	AT13-3-14-	Holocene	Sweet Bocas Trench 2	-82.27143333	9.359933333	URS	Millepora	Millepora spp	325.05
NM_2	AT13-3-14-	Holocene	Sweet Bocas Trench 2	-82.27143333	9.359933333	URS	Unknown	unknown	507.26
NM_2	AT13-3-14-	Holocene	Sweet Bocas Trench 2	-82.27143333	9.359933333	URS	Porites	Porites spp_dfp	616.30
NM_2	AT13-3-14-	Holocene	Sweet Bocas Trench 2	-82.27143333	9.359933333	URS	Acropora	Acropora cervicornis	807.18
NM_3	AT13-5-1-	Holocene	Sweet Bocas Trench 4	-82.2718	9.359983333	URS	Colpophyllia	Colpophyllia natans	27.25
NM_3	AT13-5-1-	Holocene	Sweet Bocas Trench 4	-82.2718	9.359983333	URS	Orbicella	Orbicella spp	30.95
NM_3	AT13-5-1-	Holocene	Sweet Bocas Trench 4	-82.2718	9.359983333	URS	Mussa	Mussa angulosa	281.06
NM_3	AT13-5-1-	Holocene	Sweet Bocas Trench 4	-82.2718	9.359983333	URS	Agaricia	Agaricia agaricites	0.63
NM_3	AT13-5-1-	Holocene	Sweet Bocas Trench 4	-82.2718	9.359983333	URS	Porites	Porites furcata	0.99
NM_3	AT13-5-1-	Holocene	Sweet Bocas Trench 4	-82.2718	9.359983333	URS	Agaricia	Agaricia grahamae	1.12
NM_3	AT13-5-1-	Holocene	Sweet Bocas Trench 4	-82.2718	9.359983333	URS	Agaricia	Agaricia lamarcki	1.15
NM_3	AT13-5-1-	Holocene	Sweet Bocas Trench 4	-82.2718	9.359983333	URS	Agaricia	Agaricia humilis	3.16
NM_3	AT13-5-1-	Holocene	Sweet Bocas Trench 4	-82.2718	9.359983333	URS		other not coral	3.81

NM_3	AT13-5-1-	Holocene	Sweet Bocas Trench 4	-82.2718	9.359983333	URS	Manicina	Manicina areolata	6.59
NM_3	AT13-5-1-	Holocene	Sweet Bocas Trench 4	-82.2718	9.359983333	URS	Porites	Porites spp_dfp	8.34
NM_3	AT13-5-1-	Holocene	Sweet Bocas Trench 4	-82.2718	9.359983333	URS	Favia	Favia fragum	17.89
NM_3	AT13-5-1-	Holocene	Sweet Bocas Trench 4	-82.2718	9.359983333	URS	Montastraea	Montastraea annularis	24.36
NM_3	AT13-5-1-	Holocene	Sweet Bocas Trench 4	-82.2718	9.359983333	URS	Millepora	Millepora spp	57.03
NM_3	AT13-5-1-	Holocene	Sweet Bocas Trench 4	-82.2718	9.359983333	URS	Montastraea	Montastraea franksi	133.17
NM_3	AT13-5-1-	Holocene	Sweet Bocas Trench 4	-82.2718	9.359983333	URS	Unknown	unknown	715.81
NM_3	AT13-5-1-	Holocene	Sweet Bocas Trench 4	-82.2718	9.359983333	URS	Acropora	Acropora cervicornis	1477.43
NM_4	AT13-5-2-	Holocene	Sweet Bocas Trench 4	-82.2718	9.359983333	URS	Agaricia	Agaricia fragilis	0.40
NM_4	AT13-5-2-	Holocene	Sweet Bocas Trench 4	-82.2718	9.359983333	URS	Agaricia	Agaricia agaricites	0.59
NM_4	AT13-5-2-	Holocene	Sweet Bocas Trench 4	-82.2718	9.359983333	URS	Agaricia	Agaricia undata	0.77
NM_4	AT13-5-2-	Holocene	Sweet Bocas Trench 4	-82.2718	9.359983333	URS		other not coral	1.79
NM_4	AT13-5-2-	Holocene	Sweet Bocas Trench 4	-82.2718	9.359983333	URS	Agaricia	Agaricia lamarcki	4.92
NM_4	AT13-5-2-	Holocene	Sweet Bocas Trench 4	-82.2718	9.359983333	URS	Millepora	Millepora spp	5.03
NM_4	AT13-5-2-	Holocene	Sweet Bocas Trench 4	-82.2718	9.359983333	URS	Favia	Favia fragum	7.78
NM_4	AT13-5-2-	Holocene	Sweet Bocas Trench 4	-82.2718	9.359983333	URS	Porites	Porites spp_dfp	289.06
NM_4	AT13-5-2-	Holocene	Sweet Bocas Trench 4	-82.2718	9.359983333	URS	Unknown	unknown	410.37
NM_4	AT13-5-2-	Holocene	Sweet Bocas Trench 4	-82.2718	9.359983333	URS	Acropora	Acropora palmata	615.90
NM_4	AT13-5-2-	Holocene	Sweet Bocas Trench 4	-82.2718	9.359983333	URS	Acropora	Acropora cervicornis	812.87
NM_12	AT13-5-3-	Holocene	Sweet Bocas Trench 4	-82.2718	9.359983333	URS	Montastraea	Montastraea annularis	329.87
NM_12	AT13-5-3-	Holocene	Sweet Bocas Trench 4	-82.2718	9.359983333	URS	Cladocora	Cladocora arbuscula	0.17
NM_12	AT13-5-3-	Holocene	Sweet Bocas Trench 4	-82.2718	9.359983333	URS		other not coral	1.32
NM_12	AT13-5-3-	Holocene	Sweet Bocas Trench 4	-82.2718	9.359983333	URS	Agaricia	Agaricia spp	11.42
NM_12	AT13-5-3-	Holocene	Sweet Bocas Trench 4	-82.2718	9.359983333	URS	Favia	Favia fragum	14.57
NM_12	AT13-5-3-	Holocene	Sweet Bocas Trench 4	-82.2718	9.359983333	URS	Acropora	Acropora palmata	14.64
NM_12	AT13-5-3-	Holocene	Sweet Bocas Trench 4	-82.2718	9.359983333	URS	Diploria	Diploria spp	113.10
NM_12	AT13-5-3-	Holocene	Sweet Bocas Trench 4	-82.2718	9.359983333	URS	Porites	Porites colonensis	10.69
NM_12	AT13-5-3-	Holocene	Sweet Bocas Trench 4	-82.2718	9.359983333	URS	Unknown	unknown	367.28
NM_12	AT13-5-3-	Holocene	Sweet Bocas Trench 4	-82.2718	9.359983333	URS	Porites	Porites spp_dfp	528.30
NM_12	AT13-5-3-	Holocene	Sweet Bocas Trench 4	-82.2718	9.359983333	URS	Millepora	Millepora spp	569.38
NM_12	AT13-5-3-	Holocene	Sweet Bocas Trench 4	-82.2718	9.359983333	URS	Acropora	Acropora cervicornis	1445.17
NM_12	AT13-5-3-	Holocene	Sweet Bocas Trench 4	-82.2718	9.359983333	URS	Porites	Porites astreoides	261.21
MC_8	AT13-6-1-	Holocene	Sweet Bocas Trench 5	-82.272318	9.359966667	URS	Madracis	Madracis spp	2.54
MC_8	AT13-6-1-	Holocene	Sweet Bocas Trench 5	-82.272318	9.359966667	URS	Favia	Favia fragum	3.15
MC_8	AT13-6-1-	Holocene	Sweet Bocas Trench 5	-82.272318	9.359966667	URS	Montastraea	Montastraea annularis	8.49
MC_8	AT13-6-1-	Holocene	Sweet Bocas Trench 5	-82.272318	9.359966667	URS	Manicina	Manicina areolata	10.36
MC_8	AT13-6-1-	Holocene	Sweet Bocas Trench 5	-82.272318	9.359966667	URS	Porites	Porites astreoides	39.84
MC_8	AT13-6-1-	Holocene	Sweet Bocas Trench 5	-82.272318	9.359966667	URS	Porites	Porites spp_dfp	54.26
MC_8	AT13-6-1-	Holocene	Sweet Bocas Trench 5	-82.272318	9.359966667	URS	Millepora	Millepora spp	57.12
MC_8	AT13-6-1-	Holocene	Sweet Bocas Trench 5	-82.272318	9.359966667	URS	Agaricia	Agaricia spp	133.85
MC_8	AT13-6-1-	Holocene	Sweet Bocas Trench 5	-82.272318	9.359966667	URS	Unknown	unknown	424.42
MC_8	AT13-6-1-	Holocene	Sweet Bocas Trench 5	-82.272318	9.359966667	URS	Acropora	Acropora cervicornis	1906.23
MC_9	AT13-6-2-	Holocene	Sweet Bocas Trench 5	-82.272318	9.359966667	URS	Leptoseris	Leptoseris cucullata	2.21
MC_9	AT13-6-2-	Holocene	Sweet Bocas Trench 5	-82.272318	9.359966667	URS	Cladocora	Cladocora arbuscula	0.08
MC_9	AT13-6-2-	Holocene	Sweet Bocas Trench 5	-82.272318	9.359966667	URS	Porites	Porites astreoides	0.22
MC_9	AT13-6-2-	Holocene	Sweet Bocas Trench 5	-82.272318	9.359966667	URS	Oculina	Oculina diffusa	0.52
MC_9	AT13-6-2-	Holocene	Sweet Bocas Trench 5	-82.272318	9.359966667	URS	Acropora	Acropora prolifera	1.411
MC_9	AT13-6-2-	Holocene	Sweet Bocas Trench 5	-82.272318	9.359966667	URS	Porites	Porites colonensis	1.68
MC_9	AT13-6-2-	Holocene	Sweet Bocas Trench 5	-82.272318	9.359966667	URS	Manicina	Manicina areolata	17.71
MC_9	AT13-6-2-	Holocene	Sweet Bocas Trench 5	-82.272318	9.359966667	URS	Favia	Favia fragum	33.86
MC_9	AT13-6-2-	Holocene	Sweet Bocas Trench 5	-82.272318	9.359966667	URS	Agaricia	Agaricia spp	74.62
MC_9	AT13-6-2-	Holocene	Sweet Bocas Trench 5	-82.272318	9.359966667	URS	Millepora	Millepora spp	110.61
MC_9	AT13-6-2-	Holocene	Sweet Bocas Trench 5	-82.272318	9.359966667	URS		other not coral	264.19
MC_9	AT13-6-2-	Holocene	Sweet Bocas Trench 5	-82.272318	9.359966667	URS	Acropora	Acropora cervicornis	444.03
MC_9	AT13-6-2-	Holocene	Sweet Bocas Trench 5	-82.272318	9.359966667	URS	Unknown	unknown	458.95
MC_9	AT13-6-2-	Holocene	Sweet Bocas Trench 5	-82.272318	9.359966667	URS	Porites	Porites spp_dfp	309.63
MC_9	AT13-6-2-	Holocene	Sweet Bocas Trench 5	-82.272318	9.359966667	URS	Orbicella	Orbicella spp	1701.60

NM_14	AT13-6-3-	Holocene	Sweet Bocas Trench 5	-82.272318	9.359966667	URS	Oculina	Oculina spp	0.15
NM_14	AT13-6-3-	Holocene	Sweet Bocas Trench 5	-82.272318	9.359966667	URS	cup	cup coral	0.48
NM_14	AT13-6-3-	Holocene	Sweet Bocas Trench 5	-82.272318	9.359966667	URS	Cladocora	Cladocora arbuscula	1.23
NM_14	AT13-6-3-	Holocene	Sweet Bocas Trench 5	-82.272318	9.359966667	URS	Millepora	Millepora spp	12.14
NM_14	AT13-6-3-	Holocene	Sweet Bocas Trench 5	-82.272318	9.359966667	URS	Favia	Favia fragum	15.21
NM_14	AT13-6-3-	Holocene	Sweet Bocas Trench 5	-82.272318	9.359966667	URS	Manicina	Manicina areolata	50.46
NM_14	AT13-6-3-	Holocene	Sweet Bocas Trench 5	-82.272318	9.359966667	URS	Porites	Porites colonensis	62.12
NM_14	AT13-6-3-	Holocene	Sweet Bocas Trench 5	-82.272318	9.359966667	URS	Agaricia	Agaricia spp	118.87
NM_14	AT13-6-3-	Holocene	Sweet Bocas Trench 5	-82.272318	9.359966667	URS	Orbicella	Orbicella spp	206.44
NM_14	AT13-6-3-	Holocene	Sweet Bocas Trench 5	-82.272318	9.359966667	URS	Unknown	unknown	275.95
NM_14	AT13-6-3-	Holocene	Sweet Bocas Trench 5	-82.272318	9.359966667	URS		other not coral	470.10
NM_14	AT13-6-3-	Holocene	Sweet Bocas Trench 5	-82.272318	9.359966667	URS	Porites	Porites spp_dfp	836.72
NM_14	AT13-6-3-	Holocene	Sweet Bocas Trench 5	-82.272318	9.359966667	URS	Acropora	Acropora cervicornis	947.10
MC_10	AT13-6-6-	Holocene	Sweet Bocas Trench 5	-82.272318	9.359966667	URS	Madracis	Madracis spp	0.18
MC_10	AT13-6-6-	Holocene	Sweet Bocas Trench 5	-82.272318	9.359966667	URS	Favia	Favia fragum	0.94
MC_10	AT13-6-6-	Holocene	Sweet Bocas Trench 5	-82.272318	9.359966667	URS		other not coral	2.94
MC_10	AT13-6-6-	Holocene	Sweet Bocas Trench 5	-82.272318	9.359966667	URS	Cladocora	Cladocora arbuscula	3.38
MC_10	AT13-6-6-	Holocene	Sweet Bocas Trench 5	-82.272318	9.359966667	URS	Millepora	Millepora spp	6.52
MC_10	AT13-6-6-	Holocene	Sweet Bocas Trench 5	-82.272318	9.359966667	URS	Orbicella	Orbicella spp	10.55
MC_10	AT13-6-6-	Holocene	Sweet Bocas Trench 5	-82.272318	9.359966667	URS	Agaricia	Agaricia spp	37.01
MC_10	AT13-6-6-	Holocene	Sweet Bocas Trench 5	-82.272318	9.359966667	URS	Acropora	Acropora cervicornis	109.23
MC_10	AT13-6-6-	Holocene	Sweet Bocas Trench 5	-82.272318	9.359966667	URS	Unknown	unknown	256.61
MC_10	AT13-6-6-	Holocene	Sweet Bocas Trench 5	-82.272318	9.359966667	URS	Porites	Porites spp_dfp	1269.00
MC_11	AT13-8-1-	Holocene	Sweet Bocas Trench 7	-82.27273333	9.359683333	URS	cup	cup coral	0.00
MC_11	AT13-8-1-	Holocene	Sweet Bocas Trench 7	-82.27273333	9.359683333	URS	Leptoseris	Leptoseris cucullata	0.71
MC_11	AT13-8-1-	Holocene	Sweet Bocas Trench 7	-82.27273333	9.359683333	URS		other not coral	0.82
MC_11	AT13-8-1-	Holocene	Sweet Bocas Trench 7	-82.27273333	9.359683333	URS	Oculina	Oculina diffusa	0.828
MC_11	AT13-8-1-	Holocene	Sweet Bocas Trench 7	-82.27273333	9.359683333	URS	Cladocora	Cladocora arbuscula	1.98
MC_11	AT13-8-1-	Holocene	Sweet Bocas Trench 7	-82.27273333	9.359683333	URS	Madracis	Madracis spp	2.03
MC_11	AT13-8-1-	Holocene	Sweet Bocas Trench 7	-82.27273333	9.359683333	URS	Favia	Favia fragum	4.05
MC_11	AT13-8-1-	Holocene	Sweet Bocas Trench 7	-82.27273333	9.359683333	URS	Millepora	Millepora spp	4.22
MC_11	AT13-8-1-	Holocene	Sweet Bocas Trench 7	-82.27273333	9.359683333	URS	Unknown	unknown	119.65
MC_11	AT13-8-1-	Holocene	Sweet Bocas Trench 7	-82.27273333	9.359683333	URS	Acropora	Acropora cervicornis	182.83
MC_11	AT13-8-1-	Holocene	Sweet Bocas Trench 7	-82.27273333	9.359683333	URS	Orbicella	Orbicella spp	497.31
MC_11	AT13-8-1-	Holocene	Sweet Bocas Trench 7	-82.27273333	9.359683333	URS	Porites	Porites spp_dfp	516.08
MC_11	AT13-8-1-	Holocene	Sweet Bocas Trench 7	-82.27273333	9.359683333	URS	Agaricia	Agaricia spp	1227.63
MC_14	AT13-8-2-	Holocene	Sweet Bocas Trench 7	-82.27273333	9.359683333	URS	Madracis	Madracis spp	1.42
MC_14	AT13-8-2-	Holocene	Sweet Bocas Trench 7	-82.27273333	9.359683333	URS	Cladocora	Cladocora arbuscula	2.60
MC_14	AT13-8-2-	Holocene	Sweet Bocas Trench 7	-82.27273333	9.359683333	URS	Porites	Porites astreoides	3.49
MC_14	AT13-8-2-	Holocene	Sweet Bocas Trench 7	-82.27273333	9.359683333	URS	Leptoseris	Leptoseris cucullata	3.96
MC_14	AT13-8-2-	Holocene	Sweet Bocas Trench 7	-82.27273333	9.359683333	URS		other not coral	5.62
MC_14	AT13-8-2-	Holocene	Sweet Bocas Trench 7	-82.27273333	9.359683333	URS	Millepora	Millepora spp	6.38
MC_14	AT13-8-2-	Holocene	Sweet Bocas Trench 7	-82.27273333	9.359683333	URS	unknown	unknown	88.02
MC_14	AT13-8-2-	Holocene	Sweet Bocas Trench 7	-82.27273333	9.359683333	URS	Manicina	Manicina areolata	142.29
MC_14	AT13-8-2-	Holocene	Sweet Bocas Trench 7	-82.27273333	9.359683333	URS	Porites	Porites spp_dfp	247.52
MC_14	AT13-8-2-	Holocene	Sweet Bocas Trench 7	-82.27273333	9.359683333	URS	Orbicella	Orbicella spp	320.26
MC_14	AT13-8-2-	Holocene	Sweet Bocas Trench 7	-82.27273333	9.359683333	URS	Acropora	Acropora cervicornis	468.12
MC_14	AT13-8-2-	Holocene	Sweet Bocas Trench 7	-82.27273333	9.359683333	URS	Agaricia	Agaricia spp	781.48
MC_19	AT13-9-1-	Holocene	Sweet Bocas Trench 8	-82.27365	9.360183333	URS	Leptoseris	Leptoseris cucullata	0.39
MC_19	AT13-9-1-	Holocene	Sweet Bocas Trench 8	-82.27365	9.360183333	URS	Millepora	Millepora spp	1.04
MC_19	AT13-9-1-	Holocene	Sweet Bocas Trench 8	-82.27365	9.360183333	URS	Favia	Favia fragum	1.06
MC_19	AT13-9-1-	Holocene	Sweet Bocas Trench 8	-82.27365	9.360183333	URS	Manicina	Manicina areolata	1.24
MC_19	AT13-9-1-	Holocene	Sweet Bocas Trench 8	-82.27365	9.360183333	URS	Cladocora	Cladocora arbuscula	1.41
MC_19	AT13-9-1-	Holocene	Sweet Bocas Trench 8	-82.27365	9.360183333	URS		other not coral	5.00
MC_19	AT13-9-1-	Holocene	Sweet Bocas Trench 8	-82.27365	9.360183333	URS	Eusmilia	Eusmilia fastigiata	6.80
MC_19	AT13-9-1-	Holocene	Sweet Bocas Trench 8	-82.27365	9.360183333	URS	Unknown	unknown	97.90
MC_19	AT13-9-1-	Holocene	Sweet Bocas Trench 8	-82.27365	9.360183333	URS	Agaricia	Agaricia spp	225.71

MC_19	AT13-9-1-	Holocene	Sweet Bocas Trench 8	-82.27365	9.360183333	URS	Porites	Porites spp_dfp	1621.63
MC_18	AT13-9-2-	Holocene	Sweet Bocas Trench 8	-82.27365	9.360183333	URS	cup	cup coral	0.05
MC_18	AT13-9-2-	Holocene	Sweet Bocas Trench 8	-82.27365	9.360183333	URS	Favia	Favia fragum	0.52
MC_18	AT13-9-2-	Holocene	Sweet Bocas Trench 8	-82.27365	9.360183333	URS	Cladocora	Cladocora arbuscula	2.26
MC_18	AT13-9-2-	Holocene	Sweet Bocas Trench 8	-82.27365	9.360183333	URS	Leptoseris	Leptoseris cucullata	5.30
MC_18	AT13-9-2-	Holocene	Sweet Bocas Trench 8	-82.27365	9.360183333	URS	Manicina	Manicina areolata	5.98
MC_18	AT13-9-2-	Holocene	Sweet Bocas Trench 8	-82.27365	9.360183333	URS		other not coral	11.02
MC_18	AT13-9-2-	Holocene	Sweet Bocas Trench 8	-82.27365	9.360183333	URS	Unknown	unknown	119.32
MC_18	AT13-9-2-	Holocene	Sweet Bocas Trench 8	-82.27365	9.360183333	URS	Agaricia	Agaricia spp	221.59
MC_18	AT13-9-2-	Holocene	Sweet Bocas Trench 8	-82.27365	9.360183333	URS	Porites	Porites spp_dfp	1455.73
ML_11	AT14-1-1-	Sub Recent	Casa Blanca new	-82.27989	9.36194	2.4	Madracis	Madracis asperula	6.54
ML_11	AT14-1-1-	Sub Recent	Casa Blanca new	-82.27989	9.36194	2.4	Leptoseris	Leptoseris cucullata	0.28
ML_11	AT14-1-1-	Sub Recent	Casa Blanca new	-82.27989	9.36194	2.4	Agaricia	Agaricia spp	7.75
ML_11	AT14-1-1-	Sub Recent	Casa Blanca new	-82.27989	9.36194	2.4		other not coral	124.00
ML_11	AT14-1-1-	Sub Recent	Casa Blanca new	-82.27989	9.36194	2.4	Acropora	Acropora cervicornis	125.00
ML_11	AT14-1-1-	Sub Recent	Casa Blanca new	-82.27989	9.36194	2.4	Porites	Porites spp_dfp	417.04
ML_11	AT14-1-1-	Sub Recent	Casa Blanca new	-82.27989	9.36194	2.4	Unknown	unknown	685.90
NM_7	AT14-1-2-	Sub Recent	Casa Blanca new	-82.27989	9.36194	3.1	Millepora	Millepora spp	0.46
NM_7	AT14-1-2-	Sub Recent	Casa Blanca new	-82.27989	9.36194	3.1	Madracis	Madracis decactis	0.68
NM_7	AT14-1-2-	Sub Recent	Casa Blanca new	-82.27989	9.36194	3.1	Agaricia	Agaricia spp	7.56
NM_7	AT14-1-2-	Sub Recent	Casa Blanca new	-82.27989	9.36194	3.1	Unknown	unknown	27.42
NM_7	AT14-1-2-	Sub Recent	Casa Blanca new	-82.27989	9.36194	3.1	Acropora	Acropora cervicornis	89.23
NM_7	AT14-1-2-	Sub Recent	Casa Blanca new	-82.27989	9.36194	3.1	Porites	Porites spp_dfp	92.41
NM_7	AT14-1-2-	Sub Recent	Casa Blanca new	-82.27989	9.36194	3.1		other not coral	318.73
NM_8	AT14-1-3-	Sub Recent	Casa Blanca new	-82.27989	9.36194	2.6	Madracis	Madracis asperula	2.05
NM_8	AT14-1-3-	Sub Recent	Casa Blanca new	-82.27989	9.36194	2.6	Favia	Favia fragum	0.88
NM_8	AT14-1-3-	Sub Recent	Casa Blanca new	-82.27989	9.36194	2.6	Millepora	Millepora spp	1.49
NM_8	AT14-1-3-	Sub Recent	Casa Blanca new	-82.27989	9.36194	2.6	Porites	Porites colonensis	2.04
NM_8	AT14-1-3-	Sub Recent	Casa Blanca new	-82.27989	9.36194	2.6	Eusmilia	Eusmilia fastigiata	5.06
NM_8	AT14-1-3-	Sub Recent	Casa Blanca new	-82.27989	9.36194	2.6	Agaricia	Agaricia spp	18.63
NM_8	AT14-1-3-	Sub Recent	Casa Blanca new	-82.27989	9.36194	2.6	Acropora	Acropora cervicornis	83.08
NM_8	AT14-1-3-	Sub Recent	Casa Blanca new	-82.27989	9.36194	2.6	Unknown	unknown	195.45
NM_8	AT14-1-3-	Sub Recent	Casa Blanca new	-82.27989	9.36194	2.6		other not coral	329.16
NM_8	AT14-1-3-	Sub Recent	Casa Blanca new	-82.27989	9.36194	2.6	Porites	Porites spp_dfp	999.06
NM_9_MC_2	AT14-1-7-	Sub Recent	Casa Blanca new	-82.27989	9.36194	3.9	Montastraea	Montastraea annularis	0.18
NM_9_MC_2	AT14-1-7-	Sub Recent	Casa Blanca new	-82.27989	9.36194	3.9	Oculina	Oculina spp	0.26
NM_9_MC_2	AT14-1-7-	Sub Recent	Casa Blanca new	-82.27989	9.36194	3.9	Eusmilia	Eusmilia fastigiata	0.48
NM_9_MC_2	AT14-1-7-	Sub Recent	Casa Blanca new	-82.27989	9.36194	3.9	Leptoseris	Leptoseris cucullata	1.14
NM_9_MC_2	AT14-1-7-	Sub Recent	Casa Blanca new	-82.27989	9.36194	3.9	Favia	Favia fragum	6.29
NM_9_MC_2	AT14-1-7-	Sub Recent	Casa Blanca new	-82.27989	9.36194	3.9	Madracis	Madracis spp	10.42
NM_9_MC_2	AT14-1-7-	Sub Recent	Casa Blanca new	-82.27989	9.36194	3.9	Madracis	Madracis asperula	15.66
NM_9_MC_2	AT14-1-7-	Sub Recent	Casa Blanca new	-82.27989	9.36194	3.9	Acropora	Acropora cervicornis	74.18
NM_9_MC_2	AT14-1-7-	Sub Recent	Casa Blanca new	-82.27989	9.36194	3.9	Agaricia	Agaricia spp	95.23
NM_9_MC_2	AT14-1-7-	Sub Recent	Casa Blanca new	-82.27989	9.36194	3.9	Unknown	unknown	482.48
NM_9_MC_2	AT14-1-7-	Sub Recent	Casa Blanca new	-82.27989	9.36194	3.9	Porites	Porites spp_dfp	618.86
NM_9_MC_2	AT14-1-7-	Sub Recent	Casa Blanca new	-82.27989	9.36194	3.9		other not coral	668.30
MC_3	AT14-1-8-	Sub Recent	Casa Blanca new	-82.27989	9.36194	3.4	Leptoseris	Leptoseris cucullata	0.76
MC_3	AT14-1-8-	Sub Recent	Casa Blanca new	-82.27989	9.36194	3.4	Madracis	Madracis spp	3.35
MC_3	AT14-1-8-	Sub Recent	Casa Blanca new	-82.27989	9.36194	3.4	Acropora	Acropora cervicornis	7.17
MC_3	AT14-1-8-	Sub Recent	Casa Blanca new	-82.27989	9.36194	3.4	Agaricia	Agaricia spp	10.78
MC_3	AT14-1-8-	Sub Recent	Casa Blanca new	-82.27989	9.36194	3.4		other not coral	250.10
MC_3	AT14-1-8-	Sub Recent	Casa Blanca new	-82.27989	9.36194	3.4	Porites	Porites spp_dfp	477.98
MC_3	AT14-1-8-	Sub Recent	Casa Blanca new	-82.27989	9.36194	3.4	Unknown	unknown	548.95
NM_10_MC_5	AT14-4-2-	Sub Recent	Airport Point	-82.25611	9.33611	5.2	Cladocora	Cladocora arbuscula	0.29
NM_10_MC_5	AT14-4-2-	Sub Recent	Airport Point	-82.25611	9.33611	5.2	Eusmilia	Eusmilia fastigiata	4.91
NM_10_MC_5	AT14-4-2-	Sub Recent	Airport Point	-82.25611	9.33611	5.2	Millepora	Millepora spp	0.13
NM_10_MC_5	AT14-4-2-	Sub Recent	Airport Point	-82.25611	9.33611	5.2	cup	cup coral	0.86

NM_10_MC_5	AT14-4-2-	Sub Recent	Airport Point	-82.25611	9.33611	5.2	Porites	Porites colonensis	1.01
NM_10_MC_5	AT14-4-2-	Sub Recent	Airport Point	-82.25611	9.33611	5.2	Leptoseris	Leptoseris cucullata	1.09
NM_10_MC_5	AT14-4-2-	Sub Recent	Airport Point	-82.25611	9.33611	5.2	Manicina	Manicina areolata	1.57
NM_10_MC_5	AT14-4-2-	Sub Recent	Airport Point	-82.25611	9.33611	5.2	Madracis	Madracis spp	2.27
NM_10_MC_5	AT14-4-2-	Sub Recent	Airport Point	-82.25611	9.33611	5.2	Favia	Favia fragum	3.48
NM_10_MC_5	AT14-4-2-	Sub Recent	Airport Point	-82.25611	9.33611	5.2	Oculina	Oculina spp	3.73
NM_10_MC_5	AT14-4-2-	Sub Recent	Airport Point	-82.25611	9.33611	5.2	Agaricia	Agaricia spp	437.26
NM_10_MC_5	AT14-4-2-	Sub Recent	Airport Point	-82.25611	9.33611	5.2		other not coral	551.30
NM_10_MC_5	AT14-4-2-	Sub Recent	Airport Point	-82.25611	9.33611	5.2	Unknown	unknown	606.39
NM_10_MC_5	AT14-4-2-	Sub Recent	Airport Point	-82.25611	9.33611	5.2	Porites	Porites spp_dfp	756.28
NM_11_MC_6	AT14-4-3-	Sub Recent	Airport Point	-82.25611	9.33611	5.5	Millepora	Millepora spp	1.25
NM_11_MC_6	AT14-4-3-	Sub Recent	Airport Point	-82.25611	9.33611	5.5	Porites	Porites colonensis	1.29
NM_11_MC_6	AT14-4-3-	Sub Recent	Airport Point	-82.25611	9.33611	5.5	Oculina	Oculina spp	1.60
NM_11_MC_6	AT14-4-3-	Sub Recent	Airport Point	-82.25611	9.33611	5.5	Leptoseris	Leptoseris cucullata	1.75
NM_11_MC_6	AT14-4-3-	Sub Recent	Airport Point	-82.25611	9.33611	5.5	cup	cup coral	2.20
NM_11_MC_6	AT14-4-3-	Sub Recent	Airport Point	-82.25611	9.33611	5.5	Favia	Favia fragum	3.02
NM_11_MC_6	AT14-4-3-	Sub Recent	Airport Point	-82.25611	9.33611	5.5	Eusmilia	Eusmilia fastigiata	3.19
NM_11_MC_6	AT14-4-3-	Sub Recent	Airport Point	-82.25611	9.33611	5.5	Madracis	Madracis spp	5.15
NM_11_MC_6	AT14-4-3-	Sub Recent	Airport Point	-82.25611	9.33611	5.5	Agaricia	Agaricia spp	259.50
NM_11_MC_6	AT14-4-3-	Sub Recent	Airport Point	-82.25611	9.33611	5.5	Unknown	unknown	553.38
NM_11_MC_6	AT14-4-3-	Sub Recent	Airport Point	-82.25611	9.33611	5.5	Porites	Porites spp_dfp	688.46
NM_11_MC_6	AT14-4-3-	Sub Recent	Airport Point	-82.25611	9.33611	5.5		other not coral	721.30
MC_7	AT14-4-4-	Sub Recent	Airport Point	-82.25611	9.33611	5.4	Cladocora	Cladocora arbuscula	0.19
MC_7	AT14-4-4-	Sub Recent	Airport Point	-82.25611	9.33611	5.4	cup	cup coral	1.72
MC_7	AT14-4-4-	Sub Recent	Airport Point	-82.25611	9.33611	5.4	Oculina	Oculina diffusa	1.87
MC_7	AT14-4-4-	Sub Recent	Airport Point	-82.25611	9.33611	5.4	Leptoseris	Leptoseris cucullata	2.871
MC_7	AT14-4-4-	Sub Recent	Airport Point	-82.25611	9.33611	5.4	Madracis	Madracis spp	4.64
MC_7	AT14-4-4-	Sub Recent	Airport Point	-82.25611	9.33611	5.4	Favia	Favia fragum	5.88
MC_7	AT14-4-4-	Sub Recent	Airport Point	-82.25611	9.33611	5.4	Manicina	Manicina areolata	8.18
MC_7	AT14-4-4-	Sub Recent	Airport Point	-82.25611	9.33611	5.4	Millepora	Millepora spp	9.73
MC_7	AT14-4-4-	Sub Recent	Airport Point	-82.25611	9.33611	5.4	Porites	Porites astreoides	59.79
MC_7	AT14-4-4-	Sub Recent	Airport Point	-82.25611	9.33611	5.4	Agaricia	Agaricia spp	133.09
MC_7	AT14-4-4-	Sub Recent	Airport Point	-82.25611	9.33611	5.4	Porites	Porites spp_dfp	787.39
MC_7	AT14-4-4-	Sub Recent	Airport Point	-82.25611	9.33611	5.4		other not coral	796.49
MC_7	AT14-4-4-	Sub Recent	Airport Point	-82.25611	9.33611	5.4	Unknown	unknown	889.51
NM_13	AT14-4-5-	Sub Recent	Airport Point	-82.25611	9.33611	5.1	cup	cup coral	0.12
NM_13	AT14-4-5-	Sub Recent	Airport Point	-82.25611	9.33611	5.1	Madracis	Madracis spp	0.16
NM_13	AT14-4-5-	Sub Recent	Airport Point	-82.25611	9.33611	5.1	Cladocora	Cladocora arbuscula	0.35
NM_13	AT14-4-5-	Sub Recent	Airport Point	-82.25611	9.33611	5.1	Leptoseris	Leptoseris cucullata	0.95
NM_13	AT14-4-5-	Sub Recent	Airport Point	-82.25611	9.33611	5.1	Scolymia	Scolymia cubensis	1.27
NM_13	AT14-4-5-	Sub Recent	Airport Point	-82.25611	9.33611	5.1	Porites	Porites colonensis	1.46
NM_13	AT14-4-5-	Sub Recent	Airport Point	-82.25611	9.33611	5.1	Favia	Favia fragum	7.82
NM_13	AT14-4-5-	Sub Recent	Airport Point	-82.25611	9.33611	5.1	Eusmilia	Eusmilia fastigiata	28.32
NM_13	AT14-4-5-	Sub Recent	Airport Point	-82.25611	9.33611	5.1	Oculina	Oculina spp	35.02
NM_13	AT14-4-5-	Sub Recent	Airport Point	-82.25611	9.33611	5.1	Manicina	Manicina areolata	87.14
NM_13	AT14-4-5-	Sub Recent	Airport Point	-82.25611	9.33611	5.1	Agaricia	Agaricia spp	613.5365
NM_13	AT14-4-5-	Sub Recent	Airport Point	-82.25611	9.33611	5.1		other not coral	758.90
NM_13	AT14-4-5-	Sub Recent	Airport Point	-82.25611	9.33611	5.1	Unknown	unknown	914.58
NM_13	AT14-4-5-	Sub Recent	Airport Point	-82.25611	9.33611	5.1	Porites	Porites spp_dfp	1325.18
ML_12_MC_1	AT14-4-6-	Sub Recent	Airport Point	-82.25611	9.33611	5	Leptoseris	Leptoseris cucullata	0.74
ML_12_MC_1	AT14-4-6-	Sub Recent	Airport Point	-82.25611	9.33611	5	Cladocora	Cladocora arbuscula	0.52
ML_12_MC_1	AT14-4-6-	Sub Recent	Airport Point	-82.25611	9.33611	5	cup	cup coral	0.14
ML_12_MC_1	AT14-4-6-	Sub Recent	Airport Point	-82.25611	9.33611	5	Favia	Favia fragum	2.62
ML_12_MC_1	AT14-4-6-	Sub Recent	Airport Point	-82.25611	9.33611	5	Manicina	Manicina areolata	2.87
ML_12_MC_1	AT14-4-6-	Sub Recent	Airport Point	-82.25611	9.33611	5	Madracis	Madracis spp	5.86
ML_12_MC_1	AT14-4-6-	Sub Recent	Airport Point	-82.25611	9.33611	5	Oculina	Oculina spp	5.86
ML_12_MC_1	AT14-4-6-	Sub Recent	Airport Point	-82.25611	9.33611	5	Eusmilia	Eusmilia fastigiata	7.98

ML_12_MC_1	AT14-4-6-	Sub Recent	Airport Point	-82.25611	9.33611	5	Millepora	Millepora spp	15.91
ML_12_MC_1	AT14-4-6-	Sub Recent	Airport Point	-82.25611	9.33611	5	Agaricia	Agaricia spp	341.07
ML_12_MC_1	AT14-4-6-	Sub Recent	Airport Point	-82.25611	9.33611	5	Porites	Porites spp_dfp	489.92
ML_12_MC_1	AT14-4-6-	Sub Recent	Airport Point	-82.25611	9.33611	5		other not coral	979.60
ML_12_MC_1	AT14-4-6-	Sub Recent	Airport Point	-82.25611	9.33611	5	Unknown	unknown	1523.13

References

1. Clark TR, Roff G, Zhao J-X, Feng Y-X, Done TJ, Pandolfi JM. 2014 Testing the precision and accuracy of the U–Th chronometer for dating coral mortality events in the last 100 years. *Quat. Geochronol.* **23**, 35–45.

2. Cheng H, Edwards RL, Hoff J, Gallup CD, Richards DA, Asmerom Y. 2000 The half-lives of uranium-234 and thorium-230. *Chem. Geol.* **169**, 17–33.

3. Ludwig KR. 2003 *User's Manual for Isoplot 3.00: A Geochronological Toolkit for Microsoft Excel*.

4. Toscano MA, Macintyre IG. 2003 Corrected western Atlantic sea-level curve for the last 11,000�years based on calibrated 14C dates from Acropora palmata framework and intertidal mangrove peat. *Coral Reefs* **22**, 257–270.

5. Lighty RG, Macintyre IG, Stuckenrath R. 1982 Acropora palmata reef framework: A reliable indicator of sea level in the western atlantic for the past 10,000 years. *Coral Reefs* **1**, 125–130.

6. Vogel JS, Southon JR, Nelson DE, Brown TA. 1984 Performance of catalytically condensed carbon for use in accelerator mass spectrometry. *Nucl. Instrum. Methods Phys. Res. B* **5**, 289–293.

7. In press. CALIB 14C Calibration Program. See http://calib.org (accessed on 1 December 2018).

8. Stuiver M, Reimer PJ. 1993 Extended 14C Data Base and Revised CALIB 3.0 14C Age Calibration Program. *Radiocarbon* **35**, 215–230.

9. Reimer PJ *et al.* 2013 IntCal13 and Marine13 Radiocarbon Age Calibration Curves 0–50,000 Years cal BP. *Radiocarbon* **55**, 1869–1887.

10. Wagner AJ, Guilderson TP, Slowey NC, Cole JE. 2009 Pre-Bomb Surface Water Radiocarbon of the Gulf of Mexico and Caribbean as Recorded in Hermatypic Corals. *Radiocarbon* **51**, 947–954.

11. Fredston-Hermann AL, O'Dea A, Rodriguez F, Thompson WG, Todd JA. 2013 Marked Ecological Shifts in Seagrass and Reef Molluscan Communities Since the Mid-Holocene in the Southwestern Caribbean. *Bull. Mar. Sci.* **89**, 983–1002.

12. Cramer KL, O'Dea A, Clark TR, Zhao J-X, Norris RD. 2017 Prehistorical and historical declines in Caribbean coral reef accretion rates driven by loss of parrotfish. *Nat. Commun.* **8**, 14160.
